# Atrial myxomas arise from multipotent cardiac stem cells

**DOI:** 10.1093/eurheartj/ehaa156

**Published:** 2020-04-24

**Authors:** Mariangela Scalise, Michele Torella, Fabiola Marino, Maria Ravo, Giorgio Giurato, Carla Vicinanza, Eleonora Cianflone, Teresa Mancuso, Iolanda Aquila, Luca Salerno, Giovanni Nassa, Valter Agosti, Antonella De Angelis, Konrad Urbanek, Liberato Berrino, Pierangelo Veltri, Donatella Paolino, Pasquale Mastroroberto, Marisa De Feo, Giuseppe Viglietto, Alessandro Weisz, Bernardo Nadal-Ginard, Georgina M Ellison-Hughes, Daniele Torella

**Affiliations:** Department of Experimental and Clinical Medicine, Molecular and Cellular Cardiology, Magna Graecia University, Viale Europa, 88100 Catanzaro, Italy; Department of Translational Medical Sciences, AORN dei Colli/Monaldi Hospital, University of Campania “L. Vanvitelli”, Via Leonardo Bianchi, 80131 Naples, Italy; Department of Experimental and Clinical Medicine, Molecular and Cellular Cardiology, Magna Graecia University, Viale Europa, 88100 Catanzaro, Italy; Department of Medicine, Surgery and Dentistry “Scuola Medica Salernitana”, Laboratory of Molecular Medicine and Genomics, University of Salerno, Via Salvador Allende, 84081 Baronissi (Salerno), Italy; Genomix4Life, Spin-Off of the Laboratory of Molecular Medicine and Genomics, Department of Medicine, Surgery and Dentistry, University of Salerno, Via Salvador Allende, 84081 Baronissi (Salerno), Italy; Department of Medicine, Surgery and Dentistry “Scuola Medica Salernitana”, Laboratory of Molecular Medicine and Genomics, University of Salerno, Via Salvador Allende, 84081 Baronissi (Salerno), Italy; Genomix4Life, Spin-Off of the Laboratory of Molecular Medicine and Genomics, Department of Medicine, Surgery and Dentistry, University of Salerno, Via Salvador Allende, 84081 Baronissi (Salerno), Italy; Department of Experimental and Clinical Medicine, Molecular and Cellular Cardiology, Magna Graecia University, Viale Europa, 88100 Catanzaro, Italy; Department of Experimental and Clinical Medicine, Molecular and Cellular Cardiology, Magna Graecia University, Viale Europa, 88100 Catanzaro, Italy; Department of Medical and Surgical Sciences, Magna Graecia University, Viale Europa, 88100 Catanzaro, Italy; Department of Experimental and Clinical Medicine, Molecular and Cellular Cardiology, Magna Graecia University, Viale Europa, 88100 Catanzaro, Italy; Department of Experimental and Clinical Medicine, Molecular and Cellular Cardiology, Magna Graecia University, Viale Europa, 88100 Catanzaro, Italy; Department of Experimental and Clinical Medicine, Molecular and Cellular Cardiology, Magna Graecia University, Viale Europa, 88100 Catanzaro, Italy; Department of Medicine, Surgery and Dentistry “Scuola Medica Salernitana”, Laboratory of Molecular Medicine and Genomics, University of Salerno, Via Salvador Allende, 84081 Baronissi (Salerno), Italy; Department of Experimental and Clinical Medicine, Magna Graecia University, Viale Europa, 88100 Catanzaro, Italy; Department of Experimental and Clinical Medicine, Magna Graecia University, Viale Europa, 88100 Catanzaro, Italy; Department of Experimental and Clinical Medicine, Molecular and Cellular Cardiology, Magna Graecia University, Viale Europa, 88100 Catanzaro, Italy; Department of Experimental Medicine, University of Campania “L. Vanvitelli”, Via Santa Maria di Costantinopoli, 80138 Naples, Italy; Department of Experimental Medicine, University of Campania “L. Vanvitelli”, Via Santa Maria di Costantinopoli, 80138 Naples, Italy; Department of Medical and Surgical Sciences, Magna Graecia University, Viale Europa, 88100 Catanzaro, Italy; Department of Experimental and Clinical Medicine, Magna Graecia University, Viale Europa, 88100 Catanzaro, Italy; Department of Experimental and Clinical Medicine, Magna Graecia University, Viale Europa, 88100 Catanzaro, Italy; Department of Translational Medical Sciences, AORN dei Colli/Monaldi Hospital, University of Campania “L. Vanvitelli”, Via Leonardo Bianchi, 80131 Naples, Italy; Department of Experimental and Clinical Medicine, Magna Graecia University, Viale Europa, 88100 Catanzaro, Italy; Department of Medicine, Surgery and Dentistry “Scuola Medica Salernitana”, Laboratory of Molecular Medicine and Genomics, University of Salerno, Via Salvador Allende, 84081 Baronissi (Salerno), Italy; Genomix4Life, Spin-Off of the Laboratory of Molecular Medicine and Genomics, Department of Medicine, Surgery and Dentistry, University of Salerno, Via Salvador Allende, 84081 Baronissi (Salerno), Italy; Department of Experimental and Clinical Medicine, Molecular and Cellular Cardiology, Magna Graecia University, Viale Europa, 88100 Catanzaro, Italy; Centre for Human and Applied Physiological Sciences and Centre for Stem Cells and Regenerative Medicine, School of Basic and Medical Biosciences, Faculty of Life Sciences & Medicine, King’s College London, Guys Campus - Great Maze Pond rd, SE1 1UL London, UK; Department of Experimental and Clinical Medicine, Molecular and Cellular Cardiology, Magna Graecia University, Viale Europa, 88100 Catanzaro, Italy

**Keywords:** Myxoma, Tumour histogenesis, Adult cardiac stem cells, RNASeq, MicroRNA

## Abstract

**Aims:**

Cardiac myxomas usually develop in the atria and consist of an acid-mucopolysaccharide-rich myxoid matrix with polygonal stromal cells scattered throughout. These human benign tumours are a valuable research model because of the rarity of cardiac tumours, their clinical presentation and uncertain origin. Here, we assessed whether multipotent cardiac stem/progenitor cells (CSCs) give rise to atrial myxoma tissue.

**Methods and results:**

Twenty-three myxomas were collected and analysed for the presence of multipotent CSCs. We detected myxoma cells positive for c-kit (c-kit^pos^) but very rare Isl-1 positive cells. Most of the c-kit^pos^ cells were blood lineage-committed CD45^pos^/CD31^pos^ cells. However, c-kit^pos^/CD45^neg^/CD31^neg^ cardiac myxoma cells expressed stemness and cardiac progenitor cell transcription factors. Approximately ≤10% of the c-kit^pos^/CD45^neg^/CD31^neg^ myxoma cells also expressed calretinin, a characteristic of myxoma stromal cells. *In vitro*, the c-kit^pos^/CD45^neg^/CD31^neg^ myxoma cells secrete chondroitin-6-sulfate and hyaluronic acid, which are the main components of gelatinous myxoma matrix *in vivo*. *In vitro*, c-kit^pos^/CD45^neg^/CD31^neg^ myxoma cells have stem cell properties being clonogenic, self-renewing, and sphere forming while exhibiting an abortive cardiac differentiation potential. Myxoma-derived CSCs possess a mRNA and microRNA transcriptome overall similar to normal myocardium-derived c-kit^pos^/CD45^neg^/CD31^neg^CSCs , yet showing a relatively small and relevant fraction of dysregulated mRNA/miRNAs (miR-126-3p and miR-335-5p, in particular). Importantly, myxoma-derived CSCs but not normal myocardium-derived CSCs, seed human myxoma tumours in xenograft’s in immunodeficient NOD/SCID mice.

**Conclusion:**

Myxoma-derived c-kit^pos^/CD45^neg^/CD31^neg^ CSCs fulfill the criteria expected of atrial myxoma-initiating stem cells. The transcriptome of these cells indicates that they belong to or are derived from the same lineage as the atrial multipotent c-kit^pos^/CD45^neg^/CD31^neg^ CSCs. Taken together the data presented here suggest that human myxomas could be the first-described CSC-related human heart disease.

**Figure:**
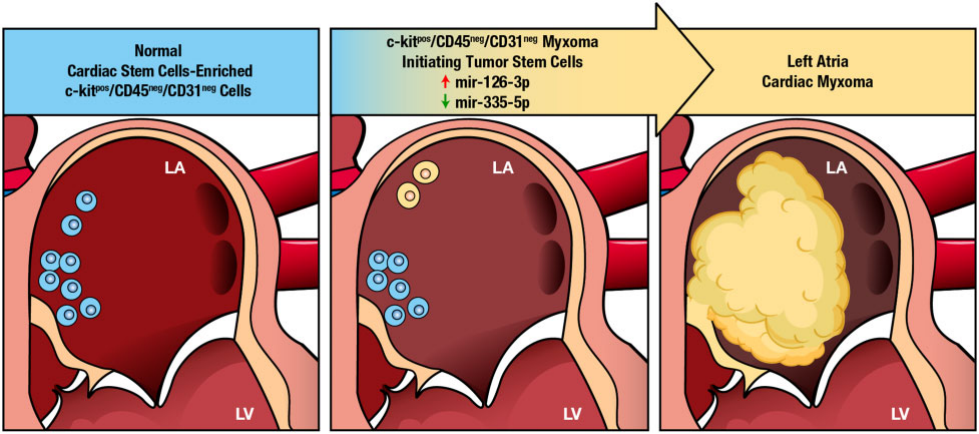

**See page 4346 for the editorial comment on this article (doi: 10.1093/eurheartj/ehaa208)**

Translational perspective

Primary tumours of the heart are rare, and among them, the vast majority is represented by cardiac myxomas. The typical cardiac myxoma is regarded as a benign neoplasm in a conventional sense. However, the oncologic designation of benignity understates the potentially clinical devastating effect this cardiac tumour may impose on the patient. Therefore, these benign tumours, despite their rarity, continue to generate interest because of their clinical presentation and uncertain histogenesis. The pathogenesis of cardiac myxoma is indeed poorly understood. Although there was debate in the past about whether cardiac myxoma was a neoplastic entity or an organized thrombus, recent gene expression and immunohistochemical studies established that cardiac myxoma is a neoplasm postulating that tumour cells arise most likely from multipotent mesenchymal cells, yet to be identified. Here, assessing 23 human cardiac myxomas, we detected within myxoma tissue c-kit^pos^/CD45^neg^/CD31^neg^ cardiac cells expressing stemness and cardiac progenitor cell transcription factors. Some (<10%) of these c-kit^pos^/CD45^neg^/CD31^neg^ cardiac myxoma cells expressed also calretinin, likely representing myxoma stromal precursor cells. More importantly, c-kit^pos^/CD45^neg^/CD31^neg^ cardiac myxoma cells secrete the typical gelatinous matrix of cardiac myxoma. These c-kit^pos^/CD45^neg^/CD31^neg^ cardiac myxoma cells show stem cell properties being clonogenic, self-renewing, and sphere forming. The myxoma-derived multipotent cells exhibit a transcriptome and miRNA network which is similar to that of the cardiac stem/progenitor cells (CSCs) from normal myocardium but with a relatively small fraction of dysregulated mRNAs and miRNAs (and miR-126-3p and miR-335-5p, in particular) involved in cell growth, differentiation and transformation pathways. Importantly, myxoma-derived multipotent CSCs seed human myxoma tumour in xenograft’s experiments in immunodeficient mice. Therefore, for the first time, we provide the evidence of the existence of a tissue-specific myxoma-initiating stem cell in atrial myxoma. Accordingly, cardiac myxomas appear to be the first CSC-related human cardiac disease.

## Introduction

Primary tumours of the heart are rare, with an incidence between 0.0017% and 0.19% in unselected patients at autopsy.^1–3^ Three quarters of the tumours are benign and nearly half the benign heart tumours are myxomas. Myxoma has an annual incidence of 0.5 per million people and most commonly presents in 30- to 50-year-old adults^4^ with a slight preponderance in women (65%).Cardiac myxomas are typically sporadic and isolated, while only in 5–10% are familial^5^ and usually develop in the atria (75% from the left atrium and 18% from the right atrium)^6^ and they are regarded as benign neoplasms in the histological conventional sense. However, the oncologic designation of benignity understates the potentially devastating effect this tumour may have on the patient. By virtue of their location, cardiac myxomas can produce myriad clinical manifestations, sometimes with fatal consequences, such as heart failure and distant embolisms of the CNS or other organs depending on their location. Therefore, these benign tumours, despite their rarity, continue to generate interest because of their clinical presentation and uncertain histogenesis.

A myxoma consists of a myxoid stroma rich in acid-mucopolysaccharide with polygonal cells scattered throughout. Differentiation of myxoma cells towards neuronal, smooth muscle, endothelial, fibroblasts, and myocytes along with typical stromal cells has been described.^7–9^ The pathogenesis of cardiac myxoma is poorly understood with early debates about whether cardiac myxomas were, indeed, neoplastic entities or organized thrombus. Recent gene expression and immunohistochemical studies have postulated that cardiac myxomas are neoplasms with the tumour cells arising most likely from multipotent mesenchymal cells yet to be identified.^10–12^ Many studies have shown that the prototypical stem-cell properties (self-renewal, clonogenicity, and multipotent differentiation ability) are relevant to some forms of cancer.^13^ ^,^ ^14^ Biologically distinct and relatively rare populations of ‘tumour-initiating’ cells have been identified in different form of cancers which have properties closely parallel to the three features that define normal stem cells. Malignant cells with these functional properties have been termed ‘cancer stem cells’ (CaSCs).

Although previously considered to be a terminally differentiated organ, the adult mammalian heart has been shown to have the ability to develop new cardiomyocytes and microvasculature throughout life.^15^ Although the extent and biological significance of this cell-renewal capacity remains controversial,^16–18^ it has been demonstrated that the adult mammalian heart harbours a population of *bona fide* resident endogenous cardiac stem/progenitor cells (CSCs). Such cells have been identified by us and other groups in a range of different mammalian species, including human. The CSCs were first characterized by expression of c-kit (also known as CD117), the kinase receptor for stem cell factor.^19^ ^,^ ^20^ However, the adult heart contains a heterogeneous c-kit^pos^ cell population most of which display blood and endothelial lineage-commitment.^21–23^ The c-kit^pos^/CD45^pos^ population shows mast cells identity, while c-kit^pos^/CD31^pos^ are endothelial (precursor) cells.^22^ ^,^ ^24^ ^,^ ^25^ Recently, it was demonstrated that only a small fraction of c-kit^pos^ cardiac cells, negative for CD45 and CD31, are enriched for multipotent CSCs.^21^ These cells are distributed throughout the myocardium with the highest density in the atria and apex.^19^ ^,^ ^26^

Based on the above, the objectives of this study were to assess whether c-kit^pos^/CD45^neg^/CD31^neg^ cardiac cells contribute to cardiac myxoma histogenesis and whether myxoma-derived c-kit^pos^/CD45^neg^/CD31^neg^ cells exhibit, *in vitro* and *in vivo*, the properties of myxoma stem cells.

## Methods

Detailed description of the methods is in the Supplementary material online, *Methods*. The authors declare that all data supporting the findings of this study are available either within the article or from the corresponding author on reasonable request.

## Results

### c-kit^pos^/CD45^neg^/CD31^neg^ cells are present within atrial myxomas and express stemness and cardiac transcription factors

**Figure 1:**
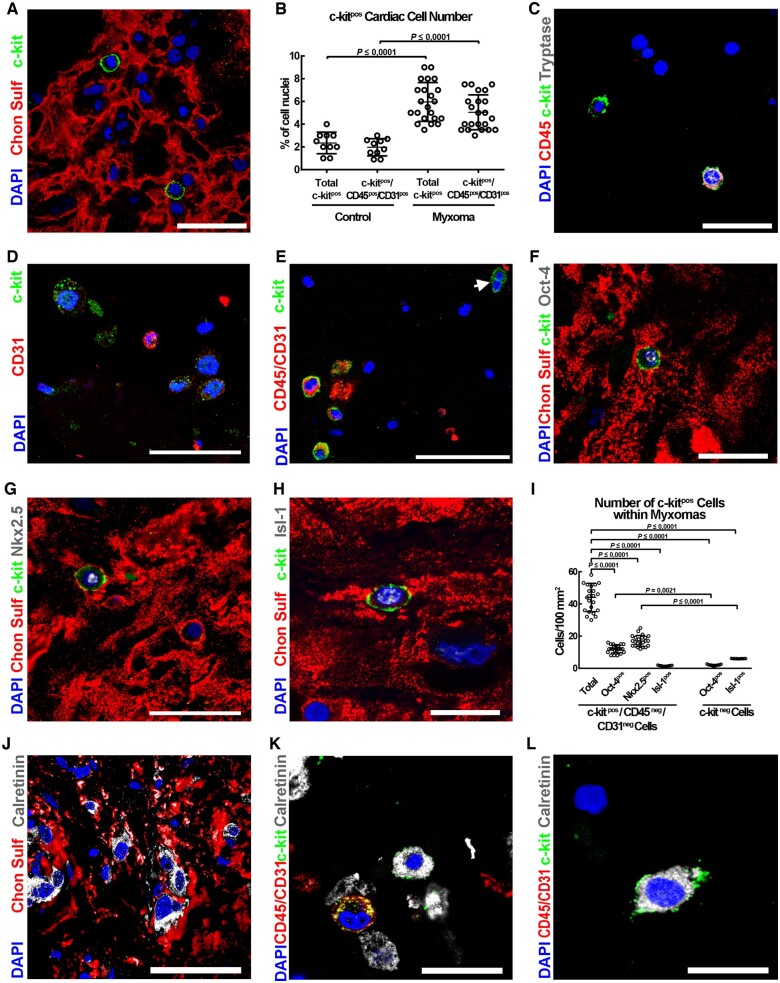
c-kit expression identifies putative multipotent resident cardiac stem cells within myxoma. (*A*) Representative confocal microscopy image of c-kit^pos^ cells within the myxoid matrix of a myxoma tumour (c-kit/green fluorescence, chondroitin sulfate/red, nuclei/DAPI/blue). Scale bar = 50 μm. (*B*) Percentage of total c-kit^pos^ cardiac cells and c-kit^pos^/CD45^pos^/CD31^pos^ cardiac cells in normal control (right atria) tissue and in myxoma tumour tissue. (*C*) Representative confocal microscopy image showing that most of the myxoma c-kit^pos^ cells were also CD45^pos^/Tryptase^pos^ (c-kit/green, CD45/red, Tryptase/white, nuclei/blue) or (*D*) CD31^pos^ (c-kit/green, CD31/red, nuclei/DAPI/blue). Scale bar = 50 μm. (*E*) Representative confocal microscopy image show that despite the majority of c-kit^pos^ cells are CD45^pos^/CD31^pos^, few of the c-kit^pos^ cells are CD45^neg^/CD31^neg^ (white arrow) in myxoma tissue. Scale bar = 50 μm. (*F*–*H*) Representative confocal microscopy images of c-kit^pos^ myxoma cells dispersed within myxoid matrix (chondroitin sulfate/red), which express stemness and cardiac progenitor cell transcription factors and respectively Oct-4 (white), Nkx2.5 (white), and Isl-1 (white). Scale bar = 50 μm. (*I*) Bar graph showing the quantification of the number of c-kit^pos^/CD45^neg^/CD31^neg^ cardiac cells, c-kit^pos^/CD45^neg^/CD31^neg^/Oct-4^pos^, c-kit^pos^/CD45^neg^/CD31^neg^/Nkx2.5^pos^, c-kit^pos^/CD45^neg^/CD31^neg^/Isl-1^pos^, c-kit^neg^/Oct-4^pos^, and c-kit^neg^/Isl-1^pos^ cardiac cells within myxoma tumour. (*J*–*L*) Representative confocal microscopy images showing the presence of the common myxoma tumour cell marker, Calretinin, dispersed in abundant myxoid matrix (*J*). Importantly, some of calretinin^pos^ cells are also c-kit^pos^ but CD45^neg^/CD31^neg^ (c-kit/green, CD45-CD31/red, calretinin/white, nuclei/DAPI/blue), which potentially identify myxoma stromal precursor cells (*K*, *L*). Scale bar =50 μm in *J*, *K*, and 20 μm in *L*. Data are presented as mean ± SD (*n* = 23 for myxoma and *n* = 10 for human controls).

Twenty-three cardiac imaging-identified myxoma tumours (Supplementary material online, *Table S1*) were surgically excised and processed for histology and immunohistochemistry analysis. All the tumours exhibited the typical histological cellular and extracellular matrix features of cardiac atrial myxoma (Supplementary material online, *Figure S1*). Dispersed within the myxoma matrix, we detected cells expressing known embryonic and adult cardiac stem/progenitor markers such as c-kit, the receptor of the stem cell factor (*Figure 1A*). Total c-kit^pos^ cardiac cells number was higher in the myxoma tissue than in the normal atrial tissue obtained from control patients (*n* = 10) (*Figure 1B*). The significance, if any, of this difference in c-kit^pos^ cells density is doubtful considering the differences in tissue composition.

We have already reported that c-kit expression in the adult myocardial cells mainly identifies endothelial and mast cells.^21^ Accordingly, most of the myxoma c-kit^pos^ cells were CD45^pos^ or CD31^pos^ (85 ± 5%) (*Figure*  1 *B*–*D*), representing either cardiac mast cells and/or endothelial cells, respectively. Moreover, myxoma c-kit^pos^/CD45^pos^ cells were also tryptase^pos^, confirming their mast cell phenotype (*Figure 1C*). A similar fraction of c-kit^pos^/CD45^pos^/CD31^pos^ cells (83 ± 7%) within the total cardiac c-kit^pos^ cells was detected in the normal right atrial tissue of the controls (*Figure 1B*). On the other hand, we also identified c-kit^pos^ cardiac myxoma cells that were negative for both CD45 and CD31 (*Figure 1E*). In the adult mammalian heart, c-kit^pos^/CD45^neg^/CD31^neg^ cells are highly enriched for CSCs.^19^ ^,^ ^21^ ^,^ ^24^ Immunofluorescent staining revealed that c-kit^pos^/CD45^neg^/CD31^neg^ myxoma cells co-express several stemness and cardiac progenitor cell transcription factors, such as Oct-4, Nkx2.5, and Isl-1 in different percentages (*Figure*  1 *F*–*I*), markers known to be also expressed in normal c-kit^pos^/CD45^neg^/CD31^neg^ CSCs from healthy human atrial tissue.^23^ ^,^ ^27^

Overall, these data raise the question of whether the c-kit^pos^/CD45^neg^/CD31^neg^ myxoma cells are derived from the atrial c-kit^pos^/CD45^neg^/CD31^neg^ stem/precursor cells and whether they are myxoma multipotent stem/progenitor cells.

### Evidence of intermediate cell phenotype between undifferentiated cardiac progenitor cells and polygonal stromal myxoma cells

The cellular compartment of myxomas is composed of stromal cells which mainly have a polygonal structure. These cells, also known as *leipidic cells*, exhibit molecular and phenotypic traits of multiple cell types, such as neuronal, endothelial, vascular, and cardiac cells. ^7^ ^,^ ^28^ ^,^ ^29^ One of the salient characteristics of cancer stem cells (CaSCs) is that they give rise to the non-CaSCs-cells within a tumour which display a more differentiated phenotype and have less proliferative capacity compared to the CaSCs. Differentiated cells within the tumour mass are believed to be the product of the progressive abortive differentiation of undifferentiated CaSCs, which involves the formation of cell intermediates constituting the cellular heterogeneity of some neoplasias.^30^ Thus, we searched for c-kit^pos^/CD45^neg^/CD31^neg^ myxoma cells expressing myxoma cell markers.

Different markers have been used to specifically identify polygonal cells.^31^ Recently, calretinin has been shown to mark the majority of myxoma cells.^32^ Most of the myxoma polygonal cells in the myxomas reported here were positive for calretinin (74 ± 7%, *Figure 1J*). Importantly, 10 ± 3% of the c-kit^pos^/CD45^neg^/CD31^neg^ myxoma cell cohort expressed calretinin (*Figure1K* and *L*). In contrast, calretinin was not expressed in c-kit^pos^ CSCs from normal atrial tissue. Thus, the presence c-kit^pos^/CD45^neg^/CD31^neg^/calretinin^pos^ cells within myxoma tissue appears to identify myxoma stromal precursor cells. This concordance potentially links the multipotent c-kit^pos^/CD45^neg^/CD31^neg^ CSCs present in the normal adult myocardium with the c-kit^pos^/CD45^neg^/CD31^neg^/calretinin^pos^ myxoma cells which are likely the precursors of the myxoma *leipidic* cells.

### Myxoma-derived c-kit^pos^/CD45^neg^/CD31^neg^ cells are clonogenic and spherogenic with an abortive cardiac differentiation potential

**Figure 2:**
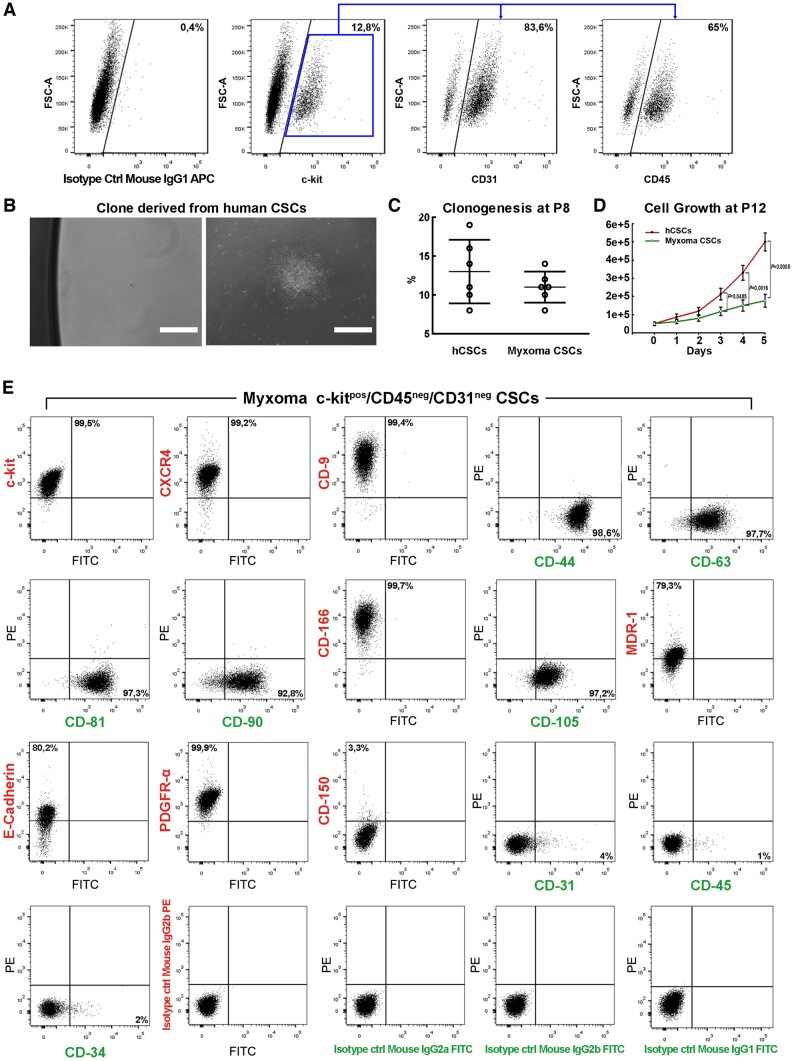
Identification, expansion/clonogenic potential and phenotipic characterization of myxoma-derived c-kit^pos^/CD45^neg^/CD31^neg^ cells* in vitro*. (*A*) Flow cytometry dot plots showing the percentage of total c-kit^pos^, c-kit^pos^/CD31^pos^, and c-kit^pos^/CD45^pos^ cardiac cells population within myxoma cells isolated from myxoma tissue (representative of *n* = 6 experiments). (*B*) Representative light microscopy images of a c-kit^pos^/CD45^neg^/CD31^neg^ hCSC clone (right) obtained depositing a single c-kit^pos^/CD45^neg^/CD31^neg^ hCSC (left) into 96-well Terasaki plates. Scale bar = 400 μm. (*C*) Graph showing the clonogenic capacity of myxoma-derived c-kit^pos^/CD45^neg^/CD31^neg^CSCs compared to normal hCSCs at P8 (representative of *n* = 6 biological replicates). (*D*) Cellular growth curve *in vitro* shows the growth deficit of c-kit^pos^/CD45^neg^/CD31^neg^ myxoma-derived CSCs compared to c-kit^pos^/CD45^neg^/CD31^neg^ hCSCs at P12. *P* < 0.05 vs. c-kit^pos^/CD45^neg^/CD31^neg^ hCSCs (representative of *n* = 6 biological replicates). (*E*) Flow cytometry dot plots showing the phenotypic characterization of c-kit^pos^/CD45^neg^/CD31^neg^ myxoma-derived ‘*putative*' CSCs. Data are presented as mean ± SD.

To assess the stem cell properties of putative multipotent myxoma stem cells,^33^ we processed the six most recently excised out of the 23 myxomas and disaggregated them to single cells by enzymatic digestion. FACS analysis confirmed that c-kit^pos^ cells were about 11 ± 3% of all nucleated cells the myxoma (*Figure 2A*). Most of these c-kit^pos^ cells co-expressed CD45 and CD31 (*Figure 2A*), confirming the immunohistochemistry data (*Figure*  1 *C* and *D*). Through MACS sorting we obtained c-kit^pos^/CD45^neg^/CD31^neg^ myxoma cells and compared their cellular properties *in vitro* to normal human CSCs similarly isolated from three right atrial appendix tissues.

Normal human CSCs are efficiently clonogenic. At passage 8 (P8), they clone (*Figure 2B*) with an efficiency of 13 ± 4% (*Figure 2C*). A typical clone of normal hCSCs has been expanded more than 50 passages so far. After these passages, these cells retain telomerase activity and normal telomere length, without cellular and/or molecular evidence of senescence (Supplementary material online, *Figure S2A*). Clonogenic normal hCSCs express mRNA and protein of recognized ‘stemness’ and cardiac progenitor genes such as *C-KIT*, *MDR-1*, *OCT-4*, *NANOG*, *BMI-1*, *TERT*, *GATA-4*, and *NKX2.5* (Supplementary material online, *Figure S2B* and *C*). The expression of genes like CD34 and CD45 is faintly detected if not practically negative (Supplementary material online, *Figure S2B*). A multimarker expression profile by flow cytometry of clonogenic normal human c-kit^pos^/CD45^neg^/CD31^neg^ CSCs shows that the membrane phenotype of these cells is positive for: CD166, CD90, CD81, CD105, CD44, CD9, CD63, CXCR4, MDR-1, E-CADHERIN, PDGFRα, and negative for: CD45, CD31, CD34, and CD150 (Supplementary material online, *Figure S2D*). When grown in suspension, normal hCSCs are multipotent and give rise to pseudoembryoid bodies, also known as cardiospheres (Supplementary material online, *Figure S3A*). When grown in specific conditioned differentiation media, normal hCSCs differentiate into cardiomyocytes, vascular smooth muscle, and endothelial cells *in vitro* (Supplementary material online, *Figure S3B* and *C*).

c-kit^pos^/CD45^neg^/CD31^neg^ cardiac myxoma-derived cells (hereafter referred to also as myxoma-derived ‘*putative*' CSCs) at P8 show a clonogenic capacity similar to normal hCSCs, 11 ± 2% (*Figure 2C*). However, at P12 the myxoma cells start to show a significant prolongation of their doubling time (*Figure 2D*) with reaches practical growth arrest at P16. This cell growth deficit was associated with a significant increase in spontaneous apoptotic cell death as revealed by TdT assay [0.1 ± 0.1% TdT^pos^ cells in normal hCSCs compared to 5 ± 3% in c-kit^pos^ myxoma cells at P16 (*P* < 0.01)]. Moreover, at P12 c-kit^pos^/CD45^neg^/CD31^neg^ myxoma-derived CSCs become larger with a diameter of 15 ± 2 μm when compared with 10 ± 2 μm of normal human eCSCs (*P* < 0.01). Interestingly, c-kit^pos^/CD45^neg^/CD31^neg^ myxoma-derived CSCs at P12 can still be cloned with an efficiency of 8 ± 2%. 

**Figure 3:**
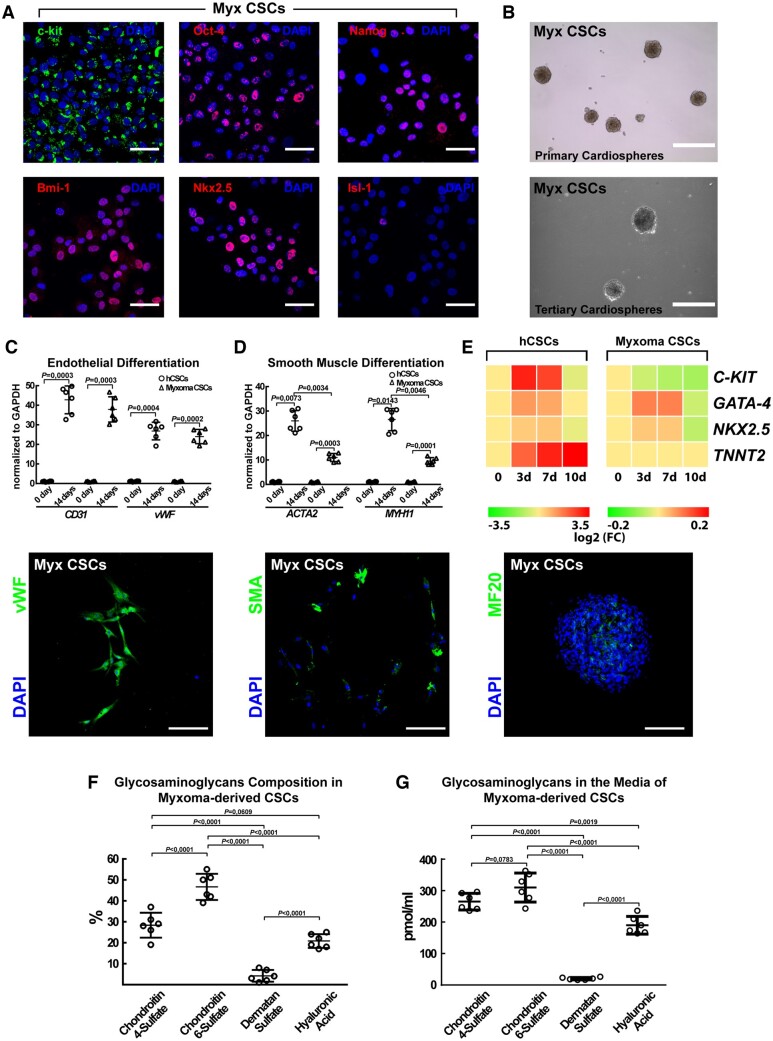
c-kit^pos^/CD45^neg^/CD31^neg^ myxoma-derived ‘*putative*' CSCs show multilineage differentiation and myxoid matrix production potential *in vitro*. (*A*) Representative confocal microscopy images obtained from cytospin preparations of c-kit^pos^/CD45^neg^/CD31^neg^ myxoma-derived CSCs revealed the expression of stemness and cardiac progenitor markers. c-kit (green), Oct-4, Nanog, Bmi-1, Nkx2.5, and Isl-1 (red) (representative of *n* = 6 biological replicates). Scale bar = 50 μm. (*B*) Representative light microscopy image of primary (top panel) and tertiary cardiospheres (bottom panel) derived from c-kit^pos^/CD45^neg^/CD31^neg^ myxoma CSCs. The number of tertiary cardiospheres is reduced in c-kit^pos^/CD45^neg^/CD31^neg^ myxoma-derived CSCs (representative of *n* = 6 biological replicates). Scale bar = 100 μm. (*C*) qRT-PCR data (upper panel) showing the expression levels of the main endothelial differentiation genes (CD31and vWf) in normal and myxoma-derived c-kit^pos^/CD45^neg^/CD31^neg^ CSCs at baseline (0 day) or after 14 days in endothelial differentiation media and representative confocal microscopy images (bottom panel) showing the expression of vWF in c-kit^pos^/CD45^neg^/CD31^neg^ myxoma-derived CSCs at 14 days after endothelial differentiation (vWf, green; nuclei/blue). Scale bar = 100 μm. (*D*) qRT-PCR data (upper panel) showing the expression levels of the main smooth muscle differentiation genes (*ACTA2* and *MYH11*) in normal and myxoma-derived c-kit^pos^/CD45^neg^/CD31^neg^ CSCs at baseline (0 day) or after 14 days in smooth muscle differentiation media and representative confocal microscopy images (bottom panel) showing the expression of smooth muscle actin (SMA) in c-kit^pos^/CD45^neg^/CD31^neg^ myxoma CSCs at 14 days after smooth muscle differentiation (SMA, green; nuclei/DAPI/blue) *P* < 0.05 vs. 0 day. Scale bar = 100 μm. (*E*) Heat maps (upper panel) showing qRT-PCR analysis of *C-KIT*, *GATA-4*, *NKX2.5*, and *TNNT2* gene transcripts in differentiating c-kit^pos^/CD45^neg^/CD31^neg^ hCSCs when compared with c-kit^pos^/CD45^neg^/CD31^neg^ myxoma-derived CSCs at different time points. Colour scale indicates change in Ct (threshold cycle) relative to the normalized GAPDH control. A representative confocal microscopy image (bottom panel) shows very few MF20^pos^ (green) cardiomyogenic-committed cells in differentiated ckit^pos^/CD45^neg^/CD31^neg^ myxoma CSC-derived Cardiospheres 14 days after cardiomyogenic differentiation. Scale bar = 100 μm (representative of *n* = 6 biological replicates). (*C*–*E*, representative of *n* = 6 biological replicates). (*F*, *G*) Bar graph showing the glicosaminoglycans composition of c-kit^pos^/CD45^neg^/CD31^neg^ myxoma-derived CSCs and in the culture media of c-kit^pos^/CD45^neg^/CD31^neg^ myxoma-derived CSCs as obtained by HPLC analysis (representative of *n* = 6 biological replicates). Data are presented as mean ± SD.

Cloned c-kit^pos^/CD45^neg^/CD31^neg^ myxoma-derived CSCs express a membrane multimarker profile (*Figure 2E*) very similar to normal human c-kit^pos^/CD45^neg^/CD31^neg^ CSCs (Supplementary material online, *Figure S2C*). Also, these clonogenic c-kit^pos^/CD45^neg^/CD31^neg^ myxoma-derived CSCs express *OCT-4*, *NANOG*, *BMI-1*, *NKX2.5*, and *ISL-*1 (*Figure 3A*) and form cardiospheres at a rate similar to normal human CSCs even though serial spherogenesis was reduced (*Figure 3B* and Supplementary material online, *Figure S3A*). When grown in specified endothelial differentiation media, clonogenic c-kit^pos^/CD45^neg^/CD31^neg^ myxoma-derived CSCs up-regulated endothelial lineage-restricted genes similarly to hCSCs (*Figure 3C* and Supplementary material online, *Figure S3B*). However, in smooth muscle cell (SMC) differentiation media, the expression of SMC lineage markers was lower in differentiated c-kit^pos^/CD45^neg^/CD31^neg^ myxoma-derived CSCs compared to normal human c-kit^pos^/CD45^neg^/CD31^neg^ CSCs (*Figure 3D* and Supplementary material online, *Figure S3B*). More importantly, when clonogenic c-kit^pos^/CD45^neg^/CD31^neg^ myxoma-derived CSCs are primed for cardiomyogenic differentiation, they up-regulated cardiac myocyte transcription factors but *in vitro* they fail to express significant levels of the contractile cardiac muscle genes , in contrast to normal hCSCs (*Figure 3E* and Supplementary material online, *Figure S3C*).

Overall, these data show that c-kit^pos^/CD45^neg^/CD31^neg^ myxoma-derived CSCs have many similarities with the normal myocardium-resident c-kit^pos^/CD45^neg^/CD31^neg^ CSCs and fulfill the *in vitro* criteria for myxoma tumour stem cells: they are clonogenic, self-renewing, and multipotent, yet they accumulate a growth deficit over passages, which is consistent with the typical low proliferation index of cardiac myxomas.^10^ ^,^ ^34^ In addition, they show a biased differentiation potential towards endothelial cells while they exhibit an abortive myogenic differentiation potential, which is also typical of myxoma tissue.^14^

### c-kit^pos^/CD45^neg^/CD31^neg^ myxoma-derived CSCs produce the typical myxoid matrix

The abundant gelatinous matrix is the most impressive aspect of the morphologic appearance of cardiac myxomas.^35–37^ This gelatinous matrix consist of glycosaminoglycans related to chondroitin sulfates and hyaluronic acid which are produced by the myxoma cells.^35–37^

Thus, we analysed and measured glycosaminoglycan content and production of myxoma-derived c-kit^pos^/CD45^neg^/CD31^neg^ CSCs at P8-12 by HPLC. Chondroitin-6-sulfate and hyaluronic acid, the main disaccharide units of glycosaminoglycans composing the gelatinous matrix,^37^ were particularly abundant in the glycosaminoglycan produced and secreted by myxoma-derived c-kit^pos^/CD45^neg^/CD31^neg^ CSCs (*Figure 3F*) and were the most prevalent in the culture medium analysed (*Figure 3G*).

These data further show that myxoma-derived c-kit^pos^/CD45^neg^/CD31^neg^ CSCs behave and show the characteristics expected from myxoma tumour stem cells.

### RNASeq analysis reveals significant similarities and differences in the transcriptome of c-kit^pos^/CD45^neg^/CD31^neg^ myxoma-derived CSCs and normal c-kit^pos^/CD45^neg^/CD31^neg^ CSCs

**Figure 4:**
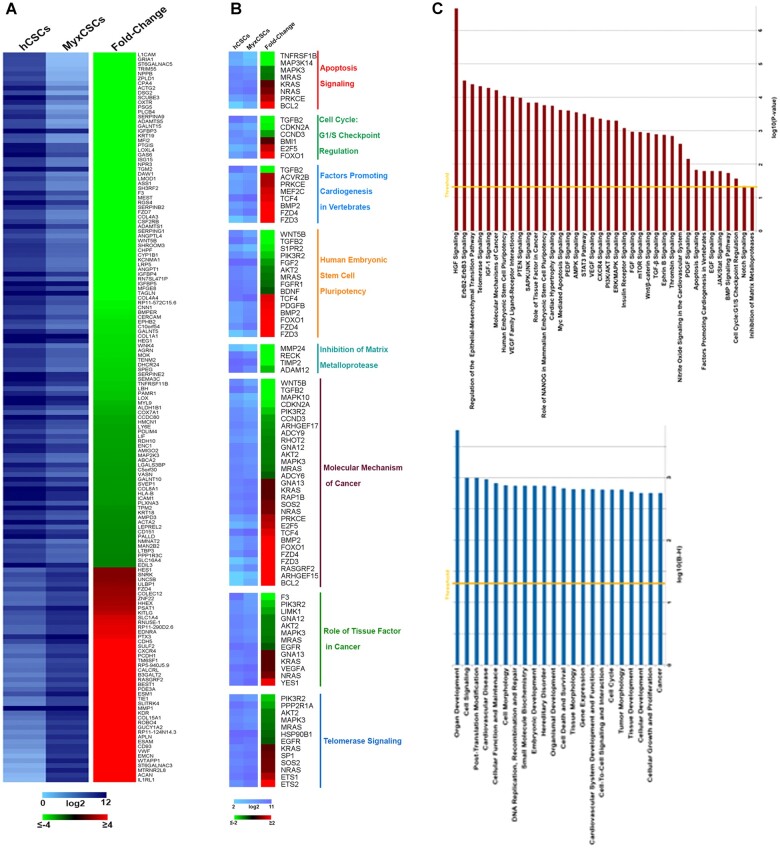
Myxoma-derived CSCs show significant and specific differences of their transcriptome when compared with control human CSCs. (*A*) Heatmaps showing the expression values, as log_2_ of normalized read-counts (left) and fold-change (FC, right) of RNAs by RNASeq analysis of hCSCs and myxoma-derived (Myx)CSCs. Only RNAs with |FC|≥3 and FDR ≤0.05 are represented. (*B*) Heatmaps showing the expression values (left) and FC of RNAs involved in selected biological functions (*P*-value ≤ 0.05) as computed by IPA in the comparison of hCSCs vs. MyxCSCs. (*C*) (Upper panel) Histogram showing the canonical pathways statistically significant (*P*-value ≤ 0.05), as defined by IPA, involving RNAs differentially expressed in the comparison of hCSCs vs. MyxCSCs. (Bottom panel) Histogram showing the biological functions statistically significant, as defined by IPA, involving RNAs differentially expressed in the comparison of hCSCs vs. MyxCSCs.

To assess the gene expression similarities and differences between the myxoma-derived ‘*putative*' CSCs and normal human CSCs, we analysed the RNA expression profile of these two-cell populations (*n* = 6 clones from 2 myxoma patients and *n* = 9 clones from 3 control human patients) by RNASeq analysis. To increase the significance of this mRNA profile analysis, only mRNAs that had a read counts ≥100 and a [fold change] ≥1.5 were considered. A total of 15 321 mRNAs were analysed in this comparison and 1243 were differentially expressed in the two cell types (see Supplementary material online, *Table S2*). That the myxoma-derived CSCs share ∼90% of gene expression with human CSCs highly point to a common origin for these cells. On the other hand, of the differentially regulated genes, 407 mRNA were significantly up-regulated (≥1.5 fold difference) and 836 were down-regulated (−1.5 fold difference) in myxoma-derived c-kit^pos^/CD45^neg^/CD31^neg^ CSCs vs. normal c-kit^pos^/CD45^neg^/CD31^neg^ CSCs (*Figure 4A* and Supplementary material online, *Table S2*).

Subsequently, we assessed the dysregulated mRNAs by bioinformatic Ingenuity pathway’s analysis (IPA) to identify the specific molecular processes affected by these mRNAs. The dysregulated mRNAs were specifically involved with apoptosis signalling, cell cycle checkpoints, factors promoting cardiogenesis, stem cell pluripotency, inhibition of matrix metalloproteases, molecular mechanism of cancer, tissue factor in cancer, and telomerase signalling (*Figure 4B* and Supplementary material online, *Table S2*). Furthermore, IPA further revealed that the dysregulated genes in myxoma-derived CSCs are components of several canonical pathways (*Figure 4C*) such as HGF and IGF-1 signalling, telomerase signalling, stem cell pluripotency, and stem cell signalling among others (*Figure 4C*). Also, these dysregulated mRNAs are involved with several specific biological functions (*Figure 4C*), such as organ development, cell signalling, and post-translational modification, cardiovascular disease, DNA replication, recombination and repair, cell cycle, and cancer among several others (*Figure 4C*).

In summary, the RNASeq analysis clearly shows that the myxoma-derived ‘*putative*' CSCs and the normal human CSCs are closely related and, most likely, the former are derived from the latter. In addition, the RNASeq data uncovered specific gene expression changes in myxoma-derived CSCs which are consistent with the cellular phenotype of myxoma tumours, with their abortive neuronal/cardiac differentiation and the hyperproduction of proteoglycans forming the prototypical myxoma gelatinous matrix. Taken together the phenotype and gene expression profile of the ‘myxoma-derived CSCs’, compared to the CSCs from normal myocardium, strongly points to them as the stem cells of cardiac myxoma.

### microRNA profile of myxoma-derived c-kit^pos^/CD45^neg^/CD31^neg^ CSCs

MicroRNAs (miRNA), small non-coding RNAs that play critical roles in normal stem cell functions in development, adult homeostasis and disease, have emerged as important regulators of tumours and cancer stem cells.^38^ Very little is yet known about microRNA regulation in the biology of adult human CSCs either *in vitro* and *in vivo*. Therefore, we assessed the microRNA expression profile in myxoma-derived c-kit^pos^/CD45^neg^/CD31^neg^ CSCs to identify miRNA/mRNA networks potentially involved in their above-described gene expression dysregulation when compared to normal human CSCs.

**Figure 5:**
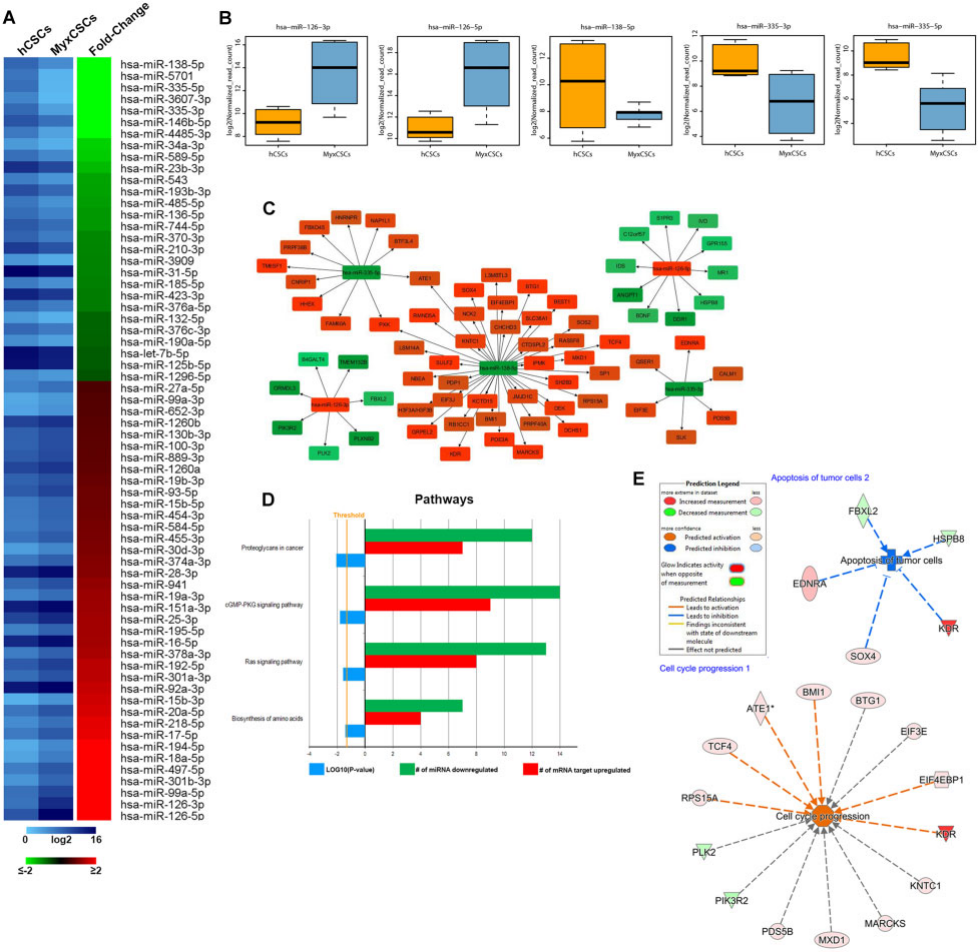
MicroRNA profile of myxoma-derived CSCs. (*A*) Heatmaps showing the expression values, expressed as log_2_ of normalized read-counts (left) and fold-change (right) of miRNAs by RNASeq analysis of hCSCs vs. MyxCSCs. Only miRNAs with |FC| ≥ 2 and FDR ≤0.05 are represented. (*B*) Boxplots showing the log_2_ normalized expression read counts of selected miRNAs in hCSCs and MyxCSCs samples. (*C*) miRNAs-mRNAs targets networks. In green are shown down-regulated miRNAs and respective regulated target mRNAs, while in red are shown up-regulated miRNAs and respective modulated target mRNAs. (*D*) Histograms showing the pathways where mRNA targets of selected miRNAs are involved (left) and the frequency of miRNAs down-regulated/mRNAs target up-regulated involved in that specific pathway in myxCSCs. (*E*) Network representation of specific biological functions where selected miRNAs/mRNA targets are involved in myxCSCs.

MicroRNA profile was obtained by RNASeq from the same samples used for the mRNA transcriptome analysis above. To increase the significance of the whole miRNA profile analysis, only miRNAs that show a read count ≥90 and a [fold-change] ≥1.5 were considered. On this basis, we detected 66 microRNA differently regulated in the comparison. Specifically, 38 miRNA up-regulated and 28 down-regulated in myxoma-derived CSCs vs. normal human CSCs (*Figure 5A* and Supplementary material online, *Table S3*). miR-138-5p is the most down-regulated in myxoma-derived CSCs (*Figure 5B* and Supplementary material online, *Table S3*). Down-regulation of miR-138 is observed in a variety of cancer types, which suggests that it may be involved in their pathogenesis.^39^ ^,^ ^40^ miR-335 was also significantly down-regulated in myxoma c-kit^pos^ cells (*Figure 5B* and Supplementary material online, *Table S3*). Interestingly, miR-335 is encoded in the mesoderm-specific transcript (*Mest*) which is significantly modulated during the differentiation of embryonic stem cells to mesodermal cardiomyocyte precursors^41^ and it is considered a potential tumour suppressor in many cancer types.^38^ Furthermore, miR-355 regulates the expression of extracellular matrix components.^41^ ^,^ ^42^

On the other hand, miR-126 is the most up-regulated in the myxoma-derived CSCs (*Figure 5B* and Supplementary material online, *Table S3*). This miR mainly contributes to normal vascular development during embryogenesis and to the regenerative function of haematopoietic stem cells.^43–45^ Recently, it has been reported that higher expression of miR-126 accelerates cancer progression by activation of mitogen-activated protein kinase and Akt, while it also suppresses tumour proliferation and tissue invasion by decreasing Crk expression. These contradictory actions suggest that miR-126 has several alternative functions specific to the type of malignancy.^46^

We specifically scanned for the known and potential mRNA targets of each down- and up-regulated miRNAs through IPA ‘microRNA Target Filter’ using ‘TargetScan Human’ and ‘TarBase’. The up-regulated mRNA putative targets of the down-regulated microRNAs in myxoma vs. normal CSCs and *vice versa* to generate the miR/mRNA networks of the two cell types (*Figure 5C*). The bioinformatic analysis revealed that the miRNA/mRNA network de-regulated in myxoma-derived CSCs fall into six main categories: proteoglycans in cancer, cGMP-PKG signalling pathway, Ras signalling pathway, biosynthesis of amino-acids, apoptosis of tumour cells, and cell cycle progression (*Figure*  5D and *E*).

Overall, the un-biased RNASeq and bioinformatics analysis of the differentially regulated miRNA networks revealed that myxoma-derived CSCs (myxoma putative stem cells) have a gene expression and regulatory microRNAs profile that is consistent and concordant with the cellular features exhibited by these cells *in vitro* and *in vivo.* Therefore, these mRNA/miRNA networks have the potential to become an entry point to unravel the main molecular mechanisms underlying the myxomatous transformation and to further ascertain the causative role of CSCs in its genesis.

### miR-126-3p and miR-335-5p regulate the biology of myxoma-derived CSCs *in vitro*

**Figure 6:**
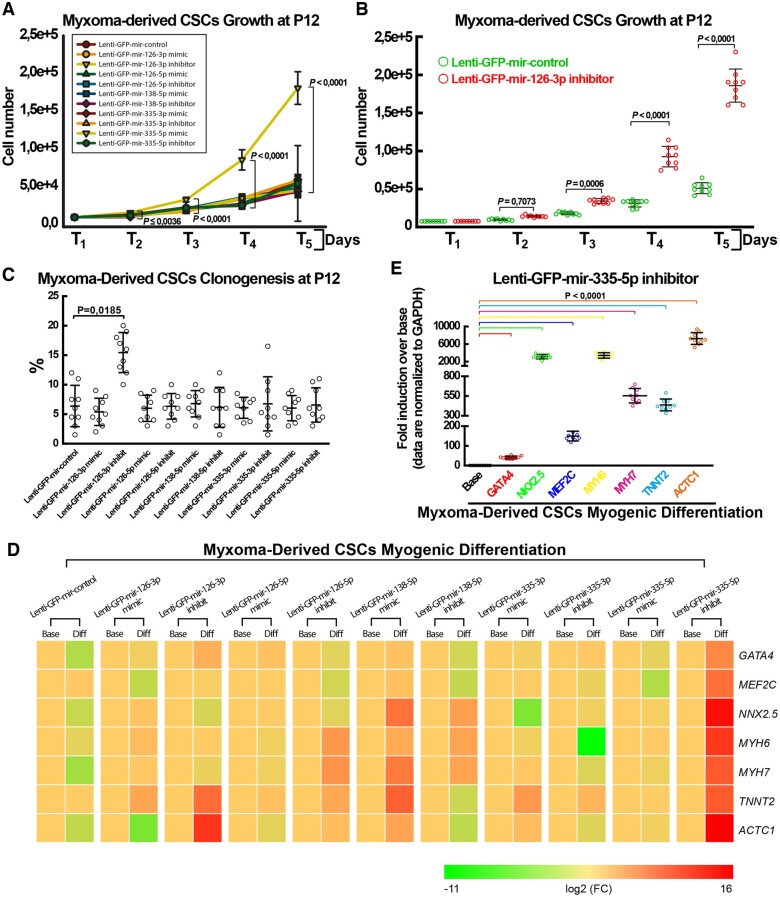
miR-126-3p and miR-335-5p dictate, respectively, the expansion/clonogenic deficit and the abortive myogenic differentiation potential of clonogenic myxoma-derived CSCs *in vitro*. (*A*) Cellular growth curve *in vitro* shows the effects of the different modulated (up-regulated by the relative miR mimic or suppressed by the relative miR inhibitor) microRNAs on myxoma-derived CSCs at P12. (*B*) Cellular growth curve *in vitro* shows that miR-126-3p suppression by Lenti-miR-126-3p inhibitor-GFP infection increases myxoma-derived CSC proliferation *in vitro* as compared to control (GFP) lentiviral infection at P12. (*C*) Graph showing the effects of the different indicated microRNA modulations on the clonogenic capacity of myxoma-derivedCSCs at P12. (*D*) Heat maps showing qRT-PCR analysis of *GATA-4*, *NKX2.5*, *MEF2C*, *MYH6*, *MYH7*, ACTC1 and *TNNT2* gene transcripts in differentiating myxoma-derived CSCs infected with the indicated Lenti-MiR or control Lenti-GFP after 14 days in cardiomyogenic media *in vitro*. Colour scale indicates change in Ct (threshold cycle) relative to the normalized GAPDH control. (*E*) qRT-PCR data showing the expression levels of the indicated cardiac genes in myxoma-derived CSCs infected with Lenti-miR-335-5p-Inhibitor after 14 days in in cardiomyogenic media *in vitro*. Data are presented as fold induction over respective baseline values (Base). All data were obtained from myxoma-derived CSCs from three different patients, where each CSC line was infected with the different indicated Lentiviral vectors. Each experiment for each CSC line was performed in triplicate. Thus, the data in each panel is representative of nine biological replicates.

On the basis of the RNASeq-based miR-nome data above, we have then evaluated the effects of the five dysregulated miRs (*Figure 5B*) on growth, clonogenic and myogenic potential of myxoma-derived CSCs (n=3) through selective gain and loss of function experiments based on the infection of lentiviral particles to transduce green fluorescent protein (GFP) together with a specific miRNA mimic or inhibitor (and a puromycin resistance cassette). At 50 MOI, we infected a large majority of myxoma-derived CSCs and with a single dose of puromycin we reached over 90% of GFP expressing cells from each specific miR-mimic and -inhibitor (Supplementary material online, *Figure S4A*). Forty-eight hours after lentiviral infection, we accordingly obtained significant overexpression of the relative miR-mimic (Supplementary material online, *Figure S4B*). More importantly, cell growth analysis of myxoma-derived CSCs shows that out of the five different miR stable overexpression or suppression, miR-126-3p inhibition was the only treatment able to rescue myxoma-derived CSCs cell growth retardation at P12 (*Figure 6A*). Indeed, miR-126-3p-suppressed myxoma-derived CSCs had a significant higher growth kinetics when compared with control Lenti-GFP infected and transduced cells (*Figure 6B*). Accordingly, clonal efficiency analysis by single-cell deposition show that miR-126-3p-inhibited myxoma-derived CSCs has higher clonogenic capacity when compared with control Lenti-GFP infected and transduced cells (*Figure 6C*). Of interest, mRNA Seq analysis of myxoma-derived cells show that miR-126-3p up-regulation in these cells when compared with normal hCSCs is associated with specular down-regulation of several genes involved with cell cycle/growth and in particular of plexin-B2 (PLXNB2) (Supplementary material online, *Figure S4C*), which has been recently shown to play a key role in growth, survival and regenerative capabilities of normal haematopoietic and leukaemic stem and progenitor cells.^47^

On the other hand, miR-335-5p inhibition was the only miR-modulating lentiviral infection that fostered robust myxoma-derived CSC myogenic commitment (*Figure 6D*). Indeed, while miR-126-3p inhibition and miR-138 up-regulation resulted in the up-regulation of single myogenic genes (*Figure 6D* and Supplementary material online, *Figure S4D*), miR-335-inhibitor treated myxoma-derived CSCs show a coherent and significant up-regulation of the main cardiac transcription factors (*GATA-4*, *MEF2C and NKX2.5,*) ensuing to an up-regulation of the main contractile genes (*MYH6*, *MYH7*, *TNNT2*, and *ACTC1*) *in vitro* upon 14 days in myogenic differentiation media (*Figure 6D*, *E* ). miR-335-5p was found down-regulated in unprimed cultured myxoma-derived CSCs when compared with human CSCs (see *Figure 5B*). Thus, miR-335-5p modulation could explain at least in part the abortive myogenic differentiation of myxoma cells. On the other hand, completely blocking miR-335-5p by a specific inhibitor could be a potential cardio-regenerative compound to enhance new myocyte formation from stem and progenitor cells and it remains mandatory to evaluate in future works the actual molecular target(s) that miR-335-p suppresses to regulate cardiomyogenic specification.

### c-kit^pos^/CD45^neg^/CD31^neg^ myxoma-derived CSCs seed myxoma tumours *in vivo*

**Figure 7:**
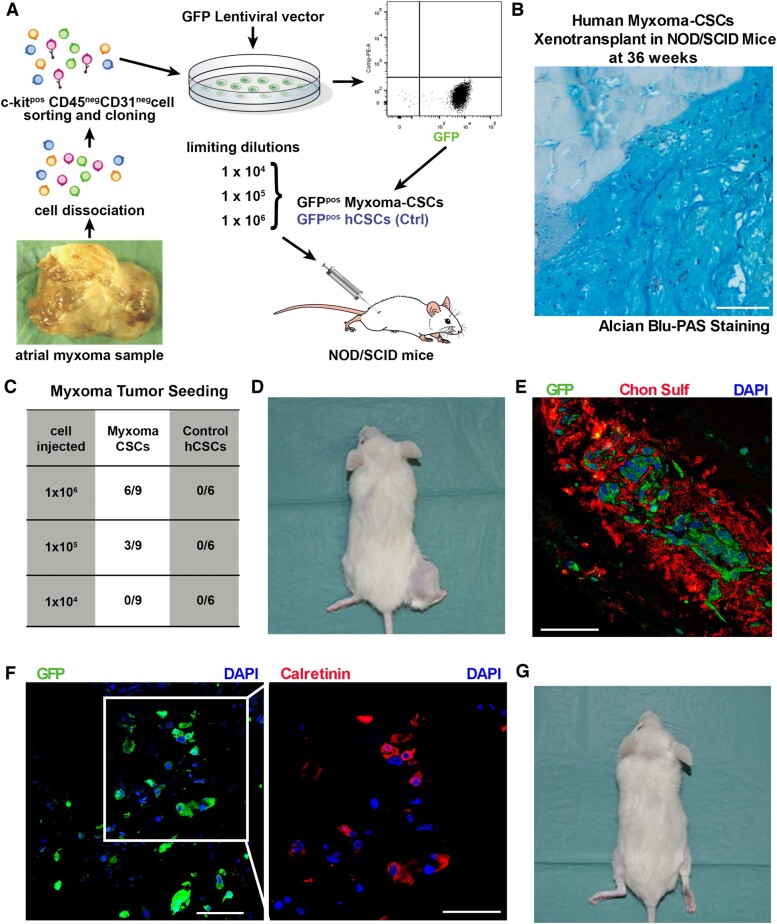
c-kit^pos^/CD45^neg^/CD31^neg^ myxoma-derived CSCs seed myxoma tumours *in vivo*. (*A*) Schematic representation of the design of the *in vivo* myxoma tumours seeding study in NOD-SCID immunodeficient mice transplanted with myxoma-derived CSCs (from three different myxoma) at different doses. Human CSCs (hCSCs) isolated from normal cardiac tissues (*n* = 3) were used as control (control hCSCs). In brief, after tissue dissociation, isolated cardiac cells were depleted of CD45^pos^ and CD31^pos^ cells then were enriched for c-kit^pos^ cardiac cells through MACS immunolabelling. Cells were kept in culture, cloned and engineered to express green fluorescent protein (GFP) by relative lentiviral transduction. GFP^pos^ myxoma-derived CSCs and GFP^pos^ normal human CSCs were transplanted into the quadriceps muscle of NOD-SCID immunodeficient mice at three different cell doses. (*B*) Representative Alcian Blue-PAS staining of the typical myxoma matrix with scattered cells formed by myxoma CSC transplantation within mouse skeletal muscle. Scale bar = 75 μm. (*C*) GFP^pos^ myxoma-derived CSCs and GFP^pos^ control hCSCs were transplanted in NOD-SCID immunodeficient mice at three different concentration. Myxoma tumour tissue formation was only detected in mice injected with GFP^pos^myxoma-derived CSCs in a dose dependent manner (*n* = 27 NOD-SCID immunodeficient mice transplanted with GFP^pos^ Myxoma-derived CSCs). No histological detectable tumour tissue or mass at any injected cell number was detected after GFP^pos^ normal human CSCs transplantation (*n* = 18 NOD-SCID immunodeficient mice transplanted with GFP^pos^ control hCSCs). (*D*) Picture of one of the only two visible and palpable myxoid matrix mass in NOD-SCID immunodeficient mice injected with 10^6^ GFP^pos^ myxoma-derived CSCs. (*E* and *F*) Representative confocal images showing scattered GFP^pos^ myxoma-derived CSCs within the myxoid matrix (*E,* Chon Sulf immunostaining, red) grown out of the transplanted GFP^pos^ myxoma-derived CSCs. Most of these GFP^pos^ cells were calretinin positive (*F*) showing that transplanted myxoma-derived CSCs differentiated *in vivo* in the typical myxoma ‘leipidic’ cells (GFP/green, Calretinin/red, nuclei/DAPI/blue). Scale bar = 150 μm (*E*, *F*), 75 μm (*F*). (*G*) Representative image of a NOD-SCID immunodeficient mouse showing no detectable or palpable tumour mass after GFP^pos^ control normal hCSCs transplantation.

Cancer-initiating (tumourigenic) cells typically form tumours after transplantation into NOD/SCID immunodeficient mice.^48^ To assess the potential of myxoma-derived c-kit^pos^/CD45^neg^/CD31^neg^ CSCs to seed cardiac myxomas *in vivo*, we transplanted 10^4^ to 10^6^ myxoma-derived CSCs (engineered to express green fluorescent protein by lentiviral transduction), isolated from three different patients, through intramuscular injection into the quadriceps muscle of 27 NOD-SCID immunodeficient mice (*Figure 7A*). Normal hCSCs (also GFP labelled) isolated from three different atrial specimens were used as controls and similarly transplanted (*Figure 7*A).

**Take home figure:**
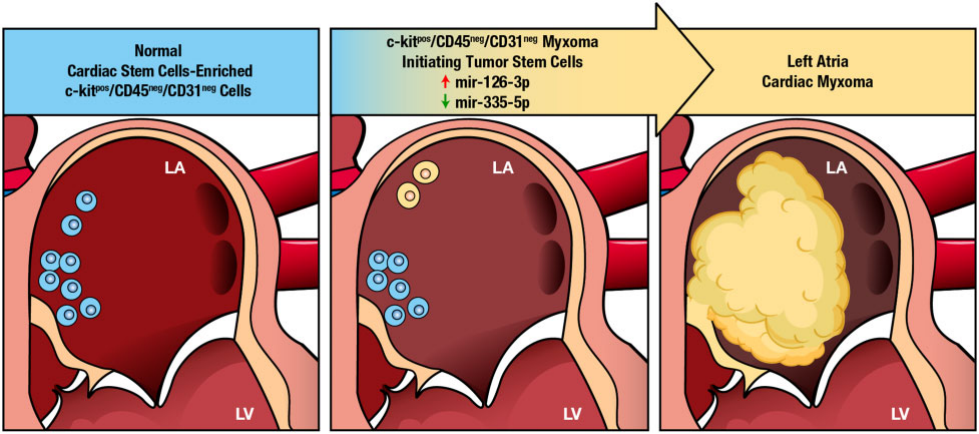
The main findings of the study showing that cardiac myxomas arise from multipotent c-kit^pos^/CD45^neg^/CD31^neg^ myxoma tumour initiating cells and the working hypothesis of their direct derivation from normal c-kit^pos^/CD45^neg^/CD31^neg^ cardiac stem/progenitor cells transformed by specific miRNA modulation.

Histologically, appearance of myxoma tumour tissue identified by Alcian Blue-PAS staining with the typical myxoma matrix with scattered cells, was directly proportional to the transplanted cell number (*Figure 7B and C*). However, palpable masses were evident only in two out of nine mice at 36 weeks after injection of 10^6^ cells (*Figure 7D*). Importantly, scattered cells within the formed myxoid matrix stained positive for GFP, establishing their derivation from the injected myxoma-derived c-kit^pos^/CD45^neg^/CD31^neg^ CSCs (*Figure 7E*). Most of these GFP cells (68 ± 5%) co-stained for Calretinin (*Figure 7F*), showing that myxoma-derived c-kit^pos^/CD45^neg^/CD31^neg^ CSCs differentiated in the typical myxoma ‘leipidic’ cells *in vivo.* In striking contrast, the c-kit^pos^/CD45^neg^/CD31^neg^ hCSCs from the normal controls were unable to form any palpable mass or any histological detectable tumour in any of the injected mice, independently of the cell number administered (*Figure 7C and G*).

Overall, the data presented in this study was strongly supportive of the notion that the myxoma-derived c-kit^pos^/CD45^neg^/CD31^neg^ multipotent cells are closely related (derived from?) the myocardial CSCs and that they are the ‘tumour-initiating cells’ of the myxoma. The results of the transplantation of these cells into immunosuppressed mice further support this notion and clearly show that these myxoma-derived c-kit^pos^/CD45^neg^/CD31^neg^ CSCs seed *de novo* myxoma tumours *in vivo* (*Take home figure*).

## Discussion

Data on mammalian and especially human cardiac neoplasias is very scarce because the myocardium is a privileged tissue which for still unknown reasons very rarely develops cancer. Therefore, information about the pathogenesis of heart neoplasias is particularly valuable to further understand its resistance to neoplastic transformation and the potential origin of those rare appearances. The main findings of the present study are: (i) c-kit^pos^/CD45^neg^/CD31^neg^ cells, which are the characteristic phenotype of the multipotent CSCs, are present within atrial myxomas and fulfill all the criteria expected for ‘myxoma initiating/stem cells’; (ii) The myxoma-derived c-kit^pos^/CD45^neg^/CD31^neg^ multipotent cell exhibit a transcriptome and miRNA network which is similar to that of the CSCs from normal myocardium but with a relatively small fraction of dysregulated mRNAs and miRNAs (miR-126-3p and miR-335, in particular) involved in cell growth, differentiation and transformation pathways; (iii) myxoma-derived initiating/stem cells *in vitro* and *in vivo* produce the typical myxoid matrix; (iv) when transplanted into immunosuppressed mice, the myxoma-derived initiating/stem cells, in contrast to the control CSCs, produce myxoma-like tumours with a polysaccharide composition, calretinin expression, and cellular distribution characteristic of the human atrial myxomas.

Cardiac myxoma is a relative rare and histologically benign gelatinous mass, which despite its benignity has significant clinical relevance due its common presentation.^3^ Indeed, myxomas can create life-threatening haemodynamic instability, due the ability of the tumour progressively or acutely to obstruct pulmonary or venous drainage, to impair flow across the atrioventricular valves causing a filling defect^6^ ^,^ ^8^ and/or embolism, due to tumour fragmentation, which typically cause cerebral embolism and transient/persistent neurological dysfunction.^6^

While clinical course of cardiac myxomas has been very precisely assessed, there is still considerable uncertainty as to their cell origin. The tissue cell composition of myxomas is typically heterogeneous, with cells expressing protein markers specific to various cell lineages including epithelial, endothelial, myogenic, myofibroblast, neural and neuro-endocrine, often within the same mass.^10^

The presence of *CSX* cardiac homeobox transcripts on its own suggested an endothelial derivation,^7^ while the detection of both *NKX2.5* and *eHAND* transcription factors within leipidic cells, suggested a myogenic cell origin^34^ and variable expression of other markers that suggest neural or neuro-endocrine origins.^34^ Therefore, it has been suggested that most likely cardiac myxomas derive from a pluripotent/multipotent mesenchymal stem cell or an endothelial progenitor cell located around the fossa ovalis and surrounding endocardium.^10^ ^,^ ^34^ Nevertheless, the precise nature of these putative stem/progenitor cell is yet unknown.

Recently, the relevant role that cells with typical stemness properties play in the development of various cancer types has been pointed out.^49^ These cells, like the stem cells of normal tissues, are characterized by their clonogenicity, multipotency, and self-renewal capacity. Malignant cells exhibiting these properties have been then defined as cancer stem cells (CaSCs) or tumour-initiating cells. Presumably, the CaSCs originate from mutations in normal stem/progenitor cells which when multiply with or without aborted differentiation induce the formation and development of some primary tumours.

Independently, various research groups have shown a regenerative process in the adult mammalian heart based, at least in part, on the existence of CSCs which can be identified and harvested from human heart tissues, as well as the different mammalian species studied.^19^ ^,^ ^21^ ^,^ ^50^ Unfortunately, ongoing research on CSC biology and regenerative potential has been jeopardized by recent scandals and controversies.^51^ ^,^ ^52^ Moreover, several genetic cell-fate mapping studies in mice have claimed that endogenous regenerative potential of CSCs is minimal.^16^ ^,^ ^53^ ^,^ ^54^ These cell-fate mapping studies have been shown to fail to test the fate of the CSCs^17^ ^,^ ^18^ ^,^ ^55^ ^,^ ^56^ and, consequently, the precise contribution of adult CSCs to endogenous cardiomyocyte replacement in homeostasis or after injury remains controversial. However, the evidence that myocardial tissue harbours cells which *in vitro* and *in vivo* are self-renewing, clonogenic, and multipotent showing CSC potential is uncontroverted.

On this basis, through cell and molecular characterizations of cardiac myxoma tissue we have shown here that these tumours harbour a cohort of c-kit^pos^/CD45^neg^/CD31^neg^ cardiac cells. A cell type which in normal mammalian tissue is highly enriched for CSCs.^21^ In myxomas, these cells are characterized by the expression of transcription factors and proteins typical of both cardiac progenitor as well as differentiated cardiac lineages. In addition, a small fraction of these cells within or isolated from the myxoma mass also express calretinin, a neuronal marker, which is highly expressed in polygonal myxoma cells.^34^ Furthermore, at early passages, the c-kit^pos^/CD45^neg^/CD31^neg^ myxoma-derived CSCs possess proliferative and clonogenic capacity similar to normal human CSCs. However, myxoma-derived CSCs, while secreting the typical mucopolysaccharides that compose the myxoma gelatinous mass *in vivo*,^34^ progressively lose their self-renewal capability and accumulate cell-cycle retardation and increased apoptotic rate through increasing passages *in vitro*. In addition, myxoma-derived CSCs exhibit an abortive differentiation potential *in vitro* whereby they undergo a multi-linage differentiation programme failing to specify into definitive cardiac cell lineages.

Although hampered by the difficulty and limitation of working with samples of human tissue which makes the data presented here mainly descriptive and falling short of being able to establish direct cause–effect relationship(s) (as it is also the case for most work based on primary human tissue), our findings strongly point to c-kit^pos^/CD45^neg^/CD31^neg^ myxoma-derived CSCs as the progeny of the CSCs present in the normal myocardium and as the initiating/stem cell of cardiac myxomas which in addition to their gelatinous structure are characterized by a low proliferative index and the presence of a heterogeneous abortively differentiated cell phenotypes. This conclusion is further strengthened by the fact that xenotransplantation of c-kit^pos^/CD45^neg^/CD31^neg^ myxoma-derived cells into immunodeficient mice seed the formation and growth of myxoma *in vivo*, highly suggesting that these cells are myxoma-initiating/stem cells.

Finally, from a clinical perspective, diagnosis of a cardiac myxoma is standardly obtained by advanced cardiac imaging^57^ ^,^ ^58^. Surrogate biomarkers (like circulating microRNAs)^59^ may help to optimize even at early stages the diagnosis and treatment modalities. Therefore, it remains highly tempting to speculate that miR-126 up-regulation in myxoma initiating CSCs, as detected here, could indeed represent a highly valuable serum biomarker for developing myxoma tumours. In addition, although surgical removal of the tumour mass is a curative therapy, in the cases of myxoma relapses, local administration of specific miR mimics/inhibitors (see *Figure 6*) to locally target c-kit^pos^/CD45 ^neg^/CD31^neg^ myxoma tumour initiating cells could be theoretically envisioned as a specific therapy for myxoma recurrences.

## Conclusions

Overall, our findings strongly support the hypothesis that cardiac myxomas are a stem cell-derived tumour arising from mesenchymal multipotent resident CSCs. Thus, it is proposed that atrial myxomas are a CSC-derived cardiac disease.

## Supplementary material

Supplementary material is available at *European Heart Journal* online.

## Funding

This work was supported by grants from the Italian Association for Cancer Research (AIRC, MFAG-2008 to D.T. and IG-23068 to A.W.), Ministry of Education, University and Research (PRIN2015 2015ZTT5KB_004 and Futuro-in-Ricerca RBFR12I3KA to D.T.), and the Italian Ministry of Health Finalized Research (GR-2010-2318945 to D.T.); Regione Campania, POR Campania FESR 2014/2020 (to A.W.); Progetto GENOMAeSALUTE (Azione 1.5; CUP: B41C17000080007 to A.W.); Progetto RarePlatNet (Azione 1.2; CUP: B63D18000380007 to A.W.) and Medical Research Council (MR/P026508/1 to GMEH).

**Conflict of interest:** none declared.

## Supplementary Material

ehaa156_Supplementary_Data
