# Supplementary material for: Atrial myxomas arise from multipotent cardiac stem cells
Source: Eur Heart J. 2020 Apr 24;41(45):4332–45. doi: 10.1093/eurheartj/ehaa156 (PMC7735815; doi:10.1093/eurheartj/ehaa156)
Supplement: ehaa156_Supplementary_Data [file ehaa156_supplementary_data.zip › ehaa156_Suppl_data/Online Materials&Methods Supplement.pdf]

ONLINE DATA SUPPLEMENT

METHODS

Patients Cohort and Samples

Myxoid and non-myxoid human tissue samples were obtained from patients undergoing heart surgery and were collected only when removal of tissue was required for surgical reasons. Collection of myxoid tissues samples was approved by the local ethics committee at the University of Campania "L. Vanvitelli" of Naples. Written informed consent was obtained from every patient before cardiac surgery. All patient data were kept anonymous. Myxoma polypoid samples, with irregular or smooth appearance, were obtained from right or left atria from patients during cardiac surgery. We examined 19 left and 4 right atrial myxomas obtained from 8 male and 15 female patients 60±10 years old. In the last 6 consecutive tumours, freshly excised samples were equally divided to be fixed for immunohistochemistry analysis or immediately processed for cell harvesting as below described.

Human myocardial samples were obtained, after written consent, from right or left atria of patients undergoing cardiac surgery at the University of Magna Graecia of Catanzaro and were used as controls (63± 4 years old). All patient data were kept anonymous.

Human CSCs and Myxoma CSCs Isolation

Both types of human samples were stored in saline on ice until ready to process (~1hr). All steps were performed at 4°C unless stated otherwise. Briefly, cardiac tissue was minced then digested with collagenase II (0.3mg/ml; Worthington Laboratories) in Dulbecco's Modified Eagle's Medium (DMEM; Sigma-Aldrich) at 37°C in a series of sequential digestions for 3 minutes each. Enzymatically released cells were filtered through a 40µm cell strainer (Becton Dickinson, BD) and collected in enzyme quenching media (DMEM + 10% FBS). The isolated cardiac cells were collected by centrifugation at 400g for 10 min, resuspended in incubation media (PBS, 0.5% BSA, 2 mM EDTA).

For the isolation of c-kit^{pos}/CD45^{neg}/CD31^{neg} CSCs, first the small cardiac cells were depleted of CD45^{pos} and CD31^{pos} cells by immunolabelling with anti-human CD45 and CD31 magnetic immunobeads (Miltenyi) diluted (1:10) in incubation media for 30 minutes at 4°C with agitation. After antibody binding, the CD45^{pos}/CD31^{pos} cells were depleted from the preparation using magnetic activated cell sorting (MACS; Miltenyi). The elution cells, which were CD45^{neg}/ CD31^{neg} were then enriched for c-kit^{pos} cardiac cells through incubation with anti-human CD117 immunobeads (Miltenyi) (1:10) for 30 minutes at 4°C with agitation and again sorted using MACS according to the manufacturer's instructions.

Cell Culture

Freshly isolated human and myxoma c-kit^{pos}/CD45^{neg}/CD31^{neg} CSCs were plated in CELL-Start (Life Technologies) coated dishes in CSC growth medium containing DMEM-F12-Ham's (Gibco, Life Technologies) with insulin-transferrin-selenium (1%, Life Technologies), epidermal growth factor (final medium concentration: 20 ng/ml, Peprotech), basal fibroblast growth factor (final medium concentration: 10 ng/ml, Peprotech) and human leukemia inhibitory factor (final medium concentration: 10 ng/ml, Miltenyi) and 1:1 ratio of Neurobasal medium (Gibco, Life Technologies) containing 37 mg of l-glutamine, B27 supplement (2%, Life Technologies) and N2 supplement (1%, Life Technologies) penicillin-streptomycin (1%, Life Technologies), Fungizone (0.1%, Life Technologies) and gentamicin (0.1%, Life Technologies). Cells were maintained in a humidified hypoxic incubator at 37°C, 5% CO_2, 2% O_2. Media were replenished every 48 hours and cells were passaged at a 1:4 ratio.

Animals and *in vivo* Tumor seeding by c-kit^{pos}/CD45^{neg}/CD31^{neg} myxoma-derived CSCs

All experiments were performed on NOD/SCID immunodeficient male mice (NOD.Cg-Prkdc^{scid} Il2rg^{tm1Wjl}/SzJ, Jackson Labs, stock number 005557). All animal experimental procedures were approved by Magna Gr*ae*cia Institutional Review Boards on Animal Use and Welfare and

performed according to the *Guide for the Care and Use of Laboratory Animals* from directive 2010/63/EU of the European Parliament. All animals received humane care, and all efforts were made to minimize animal suffering. Animals were euthanized by pentobarbital over dosage. Mice were housed under controlled conditions of 25°C, 50% relative humidity and a 12-hr light (6:00 – 18:00) and 12-hr dark cycle, with water and food (containing 18.5% protein) available *ad libitum*. Mice were anesthetized by intraperitoneal injection (i.p) of ketamine (100 mg/kg) and xylazine (5 mg/kg) or inhaled isoflurane (isoflurane 1.5% oxygen 98.5%, Iso-Vet, Healthcare).

c-kit^{pos}/CD45^{neg}/CD31^{neg} cells isolated from myxoma and normal heart tissues were cultured *in vitro,* as previously described, and engineered to express green fluorescent protein (GFP) by lentiviral transduction before transplantation. These cells were transplanted in the quadriceps muscle of NOD/SCID male mice weighing about 20 gr.

Myxoid tissue isolated from three different patients were digested to obtained three different c-kit^{pos}/CD45^{neg}/CD31^{neg}/GFP^{pos} CSC clones (n=3 clones derived from the tissue of each patient). Each clone was injected at the final concentration of $1x10^4$, $5x10^5$ and $1x10^6$ cells diluted in 200µl. Control mice were injected with cloned c-kit^{pos}/CD45^{neg}/CD31^{neg} /GFP^{pos} CSCs isolated from healthy human atrium. Muscle tissues site of cellular inoculation were collected, fixed in formalin and embedded in paraffin for histochemical and immunohistochemical analysis or stored in liquid nitrogen. The final n value for each experimental group is specified in the relative figure legends.

Histology and Immunohistochemistry

Tissue specimens were fixed and embedded in paraffin for histochemical and immunohistochemical analysis or stored in liquid nitrogen. For immunohistochemistry analysis, human tissues were cut in sections of 5µm and the slides were stained with Hematoxylin&Eosin (DAKO) to evaluate cellular architecture of the samples or with Alcian Blue (DAKO) to detect the

presence of glycosaminoglycans in myxoma samples. All images were acquired using Leica microscopy (Leica, 1128 LAS AF Software). After dewaxing in xylene and rehydration in graded concentration of ethanol, when appropriate, antigen retrieval was performed by incubating section in 10 mM sodium citrate buffer (pH 6.0) at 98°C for 30 min. Non-specific antibody binding was blocked by incubation with 10% normal donkey serum (Jackson ImmunoResearch) for 30 minutes at room temperature. Sections were incubated for 1h at room temperature with the following primary antibodies: c-kit (SantaCruz Biotechnology),Oct-4 (R&D Systems), Nkx2.5 and Isl-1 (R&D Systems) for identified cardiac progenitor cells and with antibodies against CD45 (Santa Cruz Biotechnology), CD31 (Santa CruzBiotechnology) and Tryptase (Abcam) for cardiac mast cells and endothelial cells. The stromal cells of polygonal myxoma were identified by staining with a specific antibody for Calretinin (Abcam). The myxoid matrix was detected by staining with Chondroitin Sulphate antibody (Sigma-Aldrich). In order to detect GFP^{pos} CSCs *in vivo* transplanted in NOD-SCID immunodeficient mice, anti-GFP antibody was used (Rockland). After washing in phosphate-buffered saline (PBS), sections were incubated with respective secondary antibodies (Jackson ImmunoResearch). Nuclear counterstains were performed by DAPI (4',6-diamidino-2-phenylindole, Sigma) and examined by confocal microscopy (LEICA TCS SP5 and SP8). The number of positive cells was expressed as a percent fraction of the total cells number per mm^2.

Clonogenic and Spherogenesis assay *in vitro*

To show clonogenicity, single cell cloning was employed through depositing single c-kit^{pos}/CD45^{neg}/CD31^{neg} CSCs into 96-well CELL-Start-coated Terasaki plates by serial dilution. Individual c-kit^{pos}/CD45^{neg}/CD31^{neg} CSCs were grown in CSC growth medium for 1–3 weeks when clones were identified and expanded. The clonogenicity of the c-kit^{pos}/CD45^{neg}/CD31^{neg} CSCs was determined by counting the number of wells in each 96-well plate which contained clones and expressed as a percentage. Sub-cloning was performed at every 10th passage and maintenance of

clonogenicity and stemness properties assessed. A total of 10 plates were analyzed. For cardiosphere generation, $1x10^5$ c-kit^{pos}/CD45^{neg}/CD31^{neg} CSCs were placed in bacteriological dishes with CSC growth medium. Cardiospheres were counted per plate at 14 days and the number expressed as a percentage relative to the number of plated CSCs.

Tri-Lineage differentiation potential assay *in vitro*

For vascular smooth muscle and endothelial cell commitment, cloned c-kit^{pos}/CD45^{neg} /CD31^{neg} CSCs were plated in LIF-deprived basic differentiation medium consisting of α-MEM, dexamethasone (1μM), ascorbic acid (50 μgr/ml), β-glycerophosphate (10mM) (Sigma) with 3% FBS (Invitrogen). The differentiation medium was respectively implemented with TGF-β1 (5ng/ml), all-trans retinoic acid (atRA; 10-6 mol/L, Sigma) or VEGF-A (10 ng/ml, PeproTech) for muscular or endothelial differentiation induction and complete differentiation media refreshed every 72 hours. For specific myogenic differentiation, clonogenic c-kit^{pos}/CD45^{neg}/CD31^{neg} CSC-derived cardiospheres were switched to StemPro®-34 SFM differentiation medium (a serum-free medium conditioned with StemPro®-Nutrient Supplement, Gibco, Life Technologies), Glutamine (2mM) and penicillin-streptomycin (1%, Life Technologies). For myocyte differentiation BMP4 (10ng/ml, Peprotech), Activin-A (50 ng/ml, Peprotech) , β-FGF (10ng/ml, Peprotech), Wnt-11 (150ng/ml, R&D System) and Wnt-5a (150ng/ml, R&D System). Then differentiating cardiospheres were pelleted and transferred to laminin (1μg/ml) coated dishes and Dkk-1 (150ng/ml, R&D System) was added to base differentiation medium until day 14. Cell differentiation was evaluated at 14 days.

FACS analysis

Cell analysis was performed on FACSCanto II (BD) and Fortessa X20 (BD) with FlowJo software (TREE STAR) to identify the percentage of cardiac small cells expressing different cell-surface markers of interest. A panel of different markers was used for immunophenotypic characterization of c-kit^{pos}/CD45^{neg} /CD31^{neg} myxoma CSCs and ckit^{pos}/CD45^{neg}/CD31^{neg} normal human CSCs

Specific antibodies used are shown in Online Table4. Appropriate labelled isotype controls were used to define the specific gates.

Immunocytochemistry

c-kit^{pos}/CD45^{neg}/CD31^{neg} CSCs isolated from myxoma and from human myocardial samples were grown in culture. A volume of 200 µl of a 0.3 × 10^6 cells/ml suspension were directly loaded in each cytofunnel and spin down at 800 r.p.m. for 3 min onto poly-lysine-coated slides using a Shandon Cytospin 4 Cytocentrifuge (ThermoFisher Scientific). Slides were immediately fixed using PFA 4% (Sigma-Aldrich). After fixation, cells were allowed to air dry before proceeding with immunostaining. To prepare cells for immunostaining, slides were washed with PBS, and in the case of intracellular staining, incubated in 0.1% Triton X-100:PBS at room temperature for 10 minutes. After washing with 0.1% tween:PBS and a 1 hr incubation in 10% donkey serum, cells were incubated overnight at 4°C with primary antibodies to c-kit (SantaCruz Biotechnology), Nanog (Pierce, Invitrogen),Oct4 (R&D Systems), Bmi-1(SantaCruz Biotechnology), Isl-1 and Nkx2.5 (R&D Systems), all applied at 1:50 in 0.1% Tween:PBS. Slides were then washed and incubated with corresponding secondary antibodies (Jackson Immunoresearch) for 1 hour at 37°C. The nuclear DNA of the cells was counterstained with 4',6- diamidino-2-phenylindole (DAPI) (Sigma-Aldrich) at 1µg/ml and mounted using Vectasheild mounting media (Vector labs). Imagines were acquired using a confocal microscope (LEICA TCS SP5 and SP8). A minimum of 20 random fields of view at x20 magnification were used to determine the percentage of positively stained cells per total nuclei.

Cardiospheres-derived from both ckit^{pos}/CD45^{neg}/CD31^{neg} human and myxoma CSCs were differentiated on glass chamber slides (BD Falcon) for 14 days, fixed with 4% PFA (Sigma-Aldrich) for 20 minutes and subjected to immunocytochemical staining. Cells were incubated with primary antibody against troponin (Abcam) or α sarcomeric-actin (Sigma), smooth muscle actin (Sigma-Aldrich) and vWF (Sigma-Aldrich) to evaluate the tri-lineage differentiation potential.

Then, slides were washed and incubated with corresponding secondary antibodies (Jackson Immunoresearch) for 1 hour at 37°C. The nuclear DNA of the cells was counterstained with 4',6-diamidino-2-phenylindole (DAPI) (Sigma-Aldrich) at 1µg/ml and mounted using Vectasheild mounting media (Vector labs). Specific antibodies used are shown in Online Table4.

Telomere length

Genomic DNA of human c-kit^{pos}/CD45^{neg}/Cd31^{neg} CSCs at 1th,35th and 65th passages was extracted using Quick-DNA Microprep Kit (Zymo Research). Telomere length was analyzed by using the Absolute Human Telomere Length Quantification qPCR Assay Kit (ScienCell Research Laboratories). Genomic DNA (10 ng) was amplified with the FastStart DNA Green Master (Roche Life Science) using a CFX384-Real-Time PCR System (Biorad) and data analysis was conducted according to manufacturer's instruction. For each genomic DNA samples, two different reaction were performed using two primer set: a Telomere primer set to recognize and amplify the telomere sequences and a single copy reference (SCR) primer set to normalize the data that recognizes and amplifies a 100 bp-long region on human chromosome 17, accordingly, a known telomere length as reference to calculate the telomere length of target samples. All reactions were run in triplicate. The average telomere length was calculated by following the manufacturer's instructions.

Telomerase Activity Quantification

Human c-kit^{pos}/CD45^{neg}/Cd31^{neg} CSCs at 1th,35th and 65th passages were processed according to the manufacturer's protocol using the Telomerase Activity Quantification qPCR Assay Kit (ScienCell Research Laboratories). Briefly, cell pellets were thawed in lysis reagent enables to release telomerases in the native state. Cell lysate samples were incubated with telomerase reaction buffer at 37°C for 3 hours. qRT-PCR was conducted with FastStart DNA Green Master (Roche Life Science) using a CFX384-Real-Time PCR System (Biorad) and data analysis was performed according to manufacturer's instruction. The telomere primers set recognizes and amplifies newly

synthesized telomere sequences in the assay. All reactions were run in triplicate. Telomerase activity quantification was calculated by following the manufacturer's instructions.

Lentivirus-mediated miRNA expression.

Lentivirus-vector-overexpressing or downregulating miRNAs (see Online Table 6) were generated by Abmgood and contained an expression module for GFP that enabled the detection of cells with positive infection and transduction. Lentivirus-mediated miRNA expression was conducted on Myxoma c-kit^{pos}/CD45^{neg}/Cd31^{neg} CSCs. Cells were seeded at a density of $0.5x10^5$ cells in 12-well plates and incubate at 37°C with 5% CO_2 and 3% O_2 overnight. The day after, cells were infected with lentivirus at 50 multiplicity of infection (MOI) using polybrene at a concentration of 6 μg/ml. Medium was replaced with fresh medium 24 hours after infection. 48 hours after infection the transduction efficiency was evaluated by FACS analysis through GFP expression. Infected cells were selected for the stable expression of each lentivirus vectors using puromycin (1 μg/ml) for 48 hours. Quantification of miRNA was performed in two-step RT-PCR according to the TaqMan MicroRNA Assays protocol. Briefly, total RNA was extracted by using Trizol reagent according to the manufacturer's instructions, and dissolved in RNase-free water. cDNA was reverse transcribed with 5ng of RNA using TaqMan® MicroRNA Reverse Transcription Kit, according to the manufacturer's instructions. PCR products were amplified from cDNA samples using the Taq Man® Universal PCR Master Mix. miRNAs expression was normalized to endogenous U6 snRNA expression. Primers for miRNAs were purchased from Applied Biosystems. Myxoma cells infected with lentivirus were used to evaluate miRNAs effects on cell proliferation and differentiation.

RNA extraction and RT-PCR analysis

RNA was extracted from cells c-kit^{pos}/CD45^{neg}/CD31^{neg} isolated from myxoma and from human myocardial samples, using TRIzol® RNA Isolation Reagents (Ambion) and quantitated using a Nanodrop 2000 Spectrophotometer (Thermo Fisher scientific). Reverse transcription was performed

with 0.5-1 μg of RNA using High Capacity cDNA Kit (Applied Biosystem). Quantitative RT-PCR was performed using TaqMan Primer/Probe sets (see Online Table 5) using StepOne Plus real Time PCR System (Applied Biosystems). The following genes were tested: OCT-4, NANOG, TERT, NKX2.5, GATA-4, BMI-1, CD133, C-KIT, CD34, CD31, CD45, vWf, ACTA2, MYH11, TNNT2 and MDR-1. Data were processed by the ΔCt method using StepOne Software v2.3 and mRNA was normalized to the housekeeping gene, GAPDH. All reactions were carried out in triplicate.

Myxoid matrix production by c-kit^{pos}/CD45^{neg}/CD31^{neg} Myxoma Cells

Myxoid matrix production by c-kit^{pos}/CD45^{neg}/CD31^{neg} myxoma cells were analyzed as described (2).c-kit^{pos}/CD45^{neg}/CD31^{neg} cells isolated from myxoma and fragments of tumor tissue were placed in culture separately, at 37 °C, 5% CO_2 in DMEM supplemented by 10% FBS in 6 well plates. After 7 days of culture, the medium was collected and processed. Samples of culture medium were treated with 2% NaOH overnight at 4 °C, neutralized with HCl. Were subsequently digested with Pronase and deproteinized with 10% trichloroacetic acid, and then centrifuged. The supernatant was dialyzed, and glycosaminoglycans were precipitated with cetylpyridinium chloride 0.1%, in the presence of sodium sulfate 0,012N. After centrifugation, the precipitates were washed with 95% Ethanol saturated with NaCl, then with pure ethanol and finally dried. The glycosaminoglycans were then re-suspended in water at a concentration of 1 mg/100 μl and analyzed in their composition in disaccharides.

The glycosaminoglycans of the sample solution were digested with Chondroitinase- ACII or Chondroitinase- ABC (Seikagaku Corporation, Tokyo, Japan). The samples were solubilized in 0.3 N NaOH and subsequently labeled with 1-phenyl-3- methyl-5- pirasolone (PMP) 0.5 M added to the solution, which was incubated at 70° C for 30 min. As an internal standard control xylose was used. After marking with PMP, were added to 0.1 N HCl solution for neutralization and chloroform for the extraction of disaccharides. Removed the aqueous layer, the precipitate was dissolved in water and subjected to HPLC analysis (Model L-6200, Hitachi, Japan) using columns Chemco ODS

(6x100mm). The elution was performed with a linear gradient of acetonitrile / water (1: 3, v/v) in water and 10% 200 mM phosphate buffer (pH 7.5) containing 5% acetonitrile and a flow rate of 1ml/minute at 50 °C. The peaks were detected to an absorbance of 245 nm.

RNA-Sequencing

Cell Samples

Myxoma-derived c-kit^{pos}/CD45^{neg}/CD31^{neg} cells for RNASeq analysis were taken from the first two consecutive patients from which we obtained fresh myxoma tissues. Normal CSCs were obtained from the 3 human atrial tissue controls included in the study. The 3 respective clones from each of the cell harvesting (a total of six clones for myxoma tumors vs. nine clones for normal controls) were randomly picked from the ones with highest cell density after 15 days from single cell deposition.

Libraries preparation

Libraries were prepared as described in Torella et al., (3). In detail , indexed libraries were prepared using 1 ug of total RNA as starting material, with TruSeq Stranded Total RNA Sample Prep Kit (Illumina Inc.). Libraries were sequenced (paired-end,2x100 cycles) at a concentration of 8 pM/lane on the HiSeq 2500 platform (Illumina Inc).

Bioinformatic Analysis

Bioinformatics analysis was performed as described in Tarallo et al (4). In detail, the raw sequence files generated (.fastq files) underwent quality control analysis using FASTQC (https://www.bioinformatics.babraham.ac.uk/projects/fastqc/) and quality checked reads were aligned to the human genome (assembly hg19) using TopHat version 2.0.10 (4) with the standard parameters. The number of reads mapping to each transcript was computed using HTSeq-count (4). A given RNAs was considered expressed when detected by at least ≥ I quartile of reads distribution

in each replicate (~100 raw reads) and differentially expressed RNAs were identified using DESeq2 applying a Wald's test (3). Differential expression was reported as fold-change (|FC|) ≥1.5 along with associated adjusted p-value ≤0.05. Adjusted p-values are computed according to Benjamini-Hockberg method

smallRNA-Seq

Libraries preparation

smallRNA-Seq libraries were prepared as described in Torella et al., (3). In details, libraries were generated from 120 ng using TruSeq Small RNA Sample Prep Kits (Illumina Inc.). Libraries were sequenced in single read mode, 1x50 cycles on HiSeq 2500 (Illumina Inc.).

Bioinformatics Analysis

Raw sequenced reads were analyzed using iSmaRT (5) with default parameters, using as reference miRBase 20. A given miRNAs was considered expressed when detected by at least ≥ I quartile of reads distribution in each replicate (~100 raw reads). Differentially expressed RNAs were identified using DESeq2 (5). Differential expression was reported as fold-change (|FC|) ≥1.5 along with associated adjusted p-value ≤0.05.

Target prediction

mRNAs targets of differentially expressed miRNAs were performed using IPA "microRNA Target Filter" using "TargetScan Human" and "TarBase" as source and "High Predicted" and "Experimentally Observed" as confidence values.

Functional Annotation

Functional and pathway analysis were performed using Ingenuity Pathway Analysis (IPA; Qiagen, www.ingenuity.com). Only functions and pathways shown a p-value ≤0.05 were considered.

Statistical analysis

Statistical analysis was performed with GraphPad Prism version 6.00 for Macintosh (GraphPad Software). Quantitative data are reported as mean±SE and binary data by counts. Significance between 2 groups was determined by Student's t test or paired t test as appropriate. For comparison between multiple groups, ANOVA was used. A P value <0.05 was considered significant. Bonferroni post-hoc method was used to locate the differences. In these cases, the Type 1 error ($\alpha=0.05$) was corrected by the number of statistical comparisons performed. For the *in vitro* cell and molecular biology experiments with an n=4 sample size, giving the low number of the sample, the Kruskal–Wallis test (for multiple -comparison), and the Mann group –Whitney U test (for comparison between 2 groups) were performed.

BIBLIOGRAFY

1. Xin H. Telomeric Repeat Amplification Protocol: Measuring the Activity of the Telomerase. In: Humana Press, 2011:107–111.

2. Negishi M, Sakamoto H, Sakamaki T, et al. Disaccharide analysis of glycosaminoglycans synthesized by cardiac myxoma cells in tumor tissues and in cell culture. Life Sci. 2003 Jul 4;73(7):849-56.

3. Torella D, Iaconetti C, Tarallo R, et al. MiRNA regulation of the hyperproliferative phenotype of vascular smooth muscle cells in diabetes. Diabetes 2018;67.

4. Tarallo R, Giurato G, Bruno G, et al. The nuclear receptor ERβ engages AGO2 in regulation of gene transcription, RNA splicing and RISC loading. Genome Biol 2017;18:189.

5. Panero R, Rinaldi A, Memoli D, et al. iSmaRT: a toolkit for a comprehensive analysis of small RNA-Seq data. Bioinformatics 2017;33:btw734.
