# Supplementary material for: Atrial myxomas arise from multipotent cardiac stem cells
Source: Eur Heart J. 2020 Apr 24;41(45):4332–45. doi: 10.1093/eurheartj/ehaa156 (PMC7735815; doi:10.1093/eurheartj/ehaa156)
Supplement: ehaa156_Supplementary_Data [file ehaa156_supplementary_data.zip › ehaa156_Suppl_data/Online Supplementary Figures 1-4.pdf]

Online Supplementary Figure 1

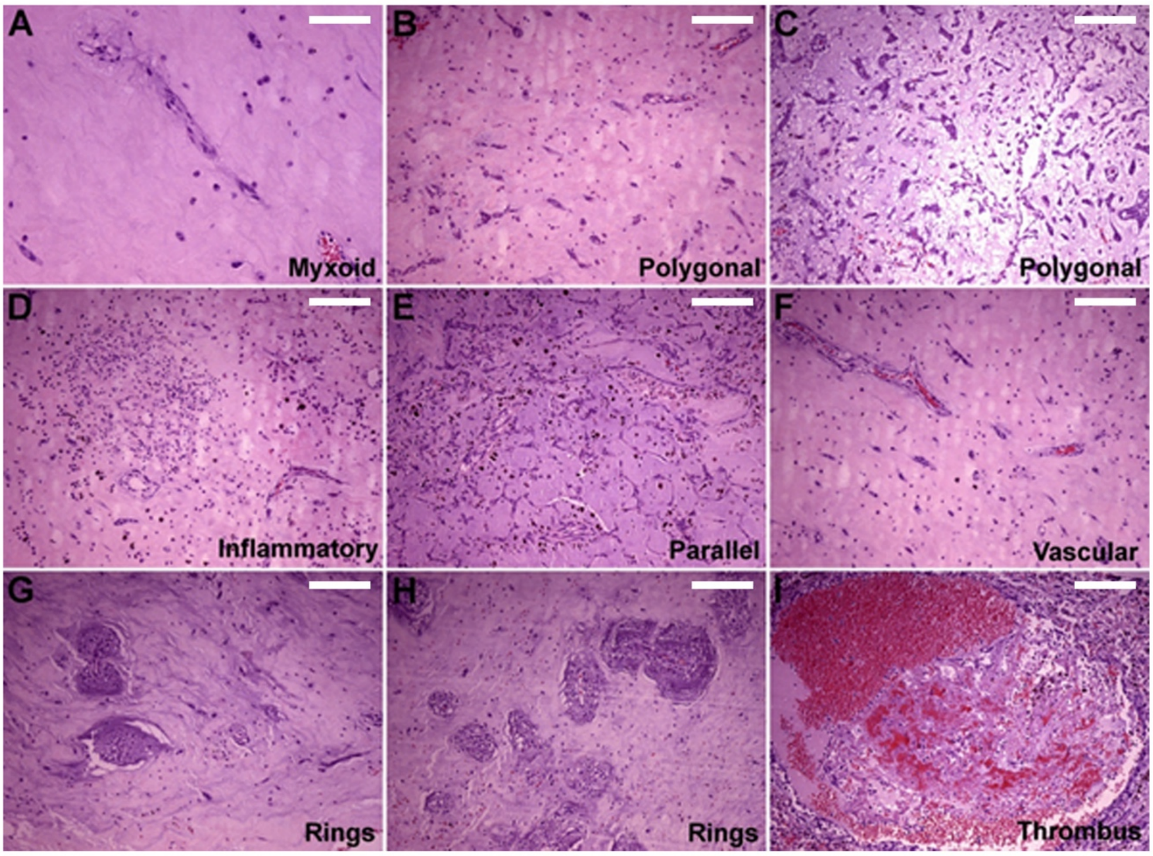

Fig. S1. Histological Features of Myxoma Tumors
(A-I) Histology microscopy images of atrial myxoma samples showing the typical abundant myxoid matrix with stellate cells characterized by eosinophilic cytoplasm, indistinct cell borders and elongated polygonal cells. Inflammatory cells are also present. Myxoma cells form rings, cords and nests throughout tumor are also shown. Moreover, the presence of extravasated erythrocytes and hemosiderin deposition determine rare thrombus formation. Scale bar=100µm (A) and 200µm (B-I).

Online Supplementary Figure 2

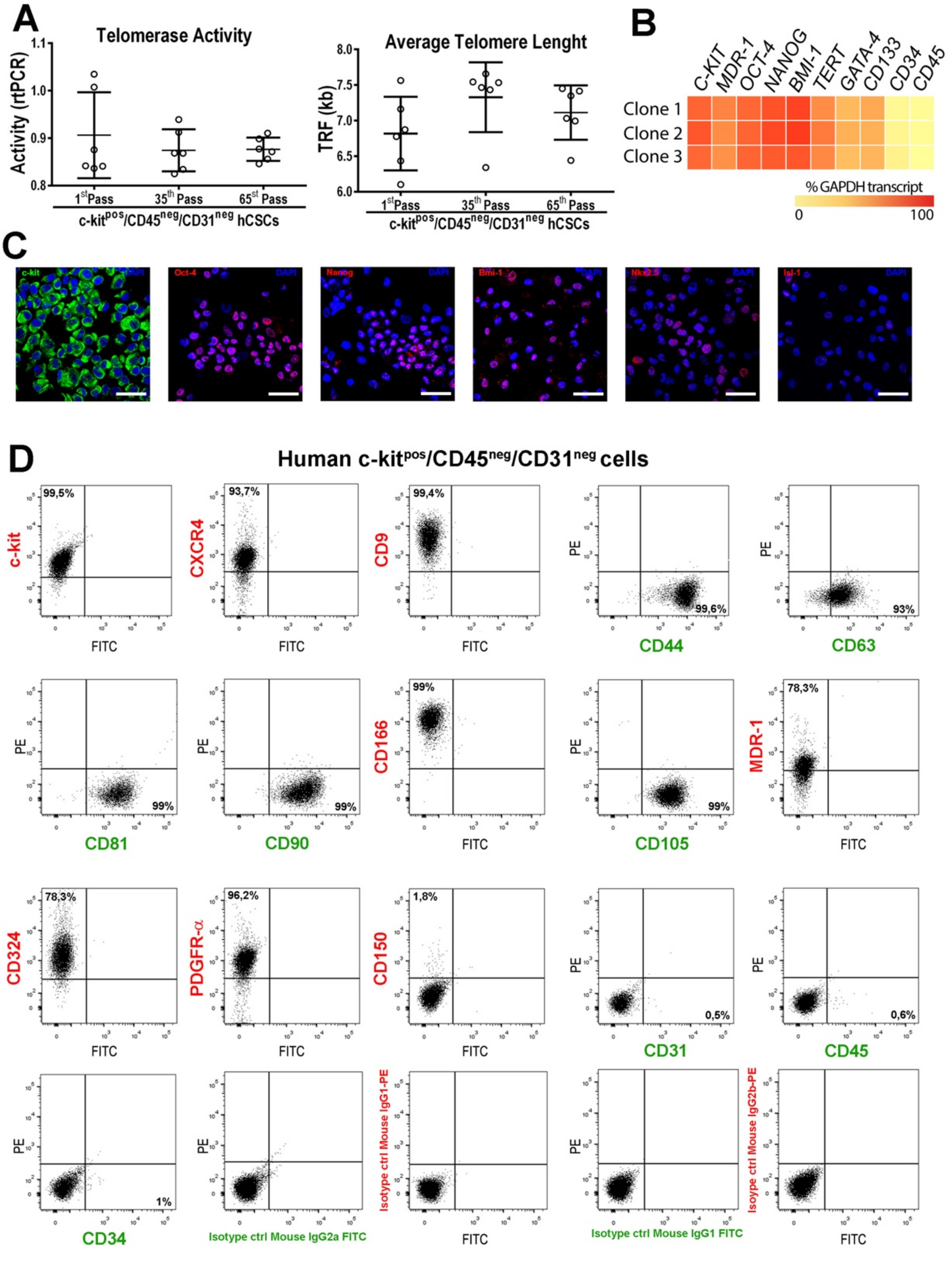

Fig. S2. Growth potential and phenotypic characterization of human c-kit^{pos}/CD45^{neg}/CD31^{neg} CSCs (A) Left graph shows TERT activity measured by '*Telomerase Activity Quantification qPCR assay kit*' in c-kit^{pos}/CD45^{neg}/CD31^{pos} hCSCs at the indicated passages. Clonal c-kit^{pos} /CD45^{neg}/CD31^{neg} hCSCs maintain TERT expression even after 50 passages in culture. Right graph shows that clonal c-kit^{pos} /CD45^{neg}/CD31^{neg} hCSCs preserve over time normal telomere length. Data are mean±S.D. **(B)** Heat map showing qRT-PCR analysis of three different clones derived from normal c-kit^{pos} hCSCs. Three clones maintain a similar expression of multipotent and cardiac transcription factor genes. Color scale indicates change in Ct (threshold cycle) relative to the normalized GAPDH control (Representative of n=6 biological replicates). **(C)** Representative

confocal microscopy images from cytospin preparations of c-kit^{pos}/CD45^{neg}/CD31^{neg} hCSCs showing the expression of stemness and cardiac progenitor markers, c-kit (green), Oct-4, Nanog, Bmi-1, Nkx2.5, Isl-1 (red). Scale bar=100µm. (Representative of n=6 biological replicates). **(D)** Flow cytometry dot plots showing the specific membrane phenotype of typical clonogenic c-kit^{pos}/CD45^{neg}/CD31^{neg} hCSCs. (Representative of n=6 experiments).

Supplementary Figure 3

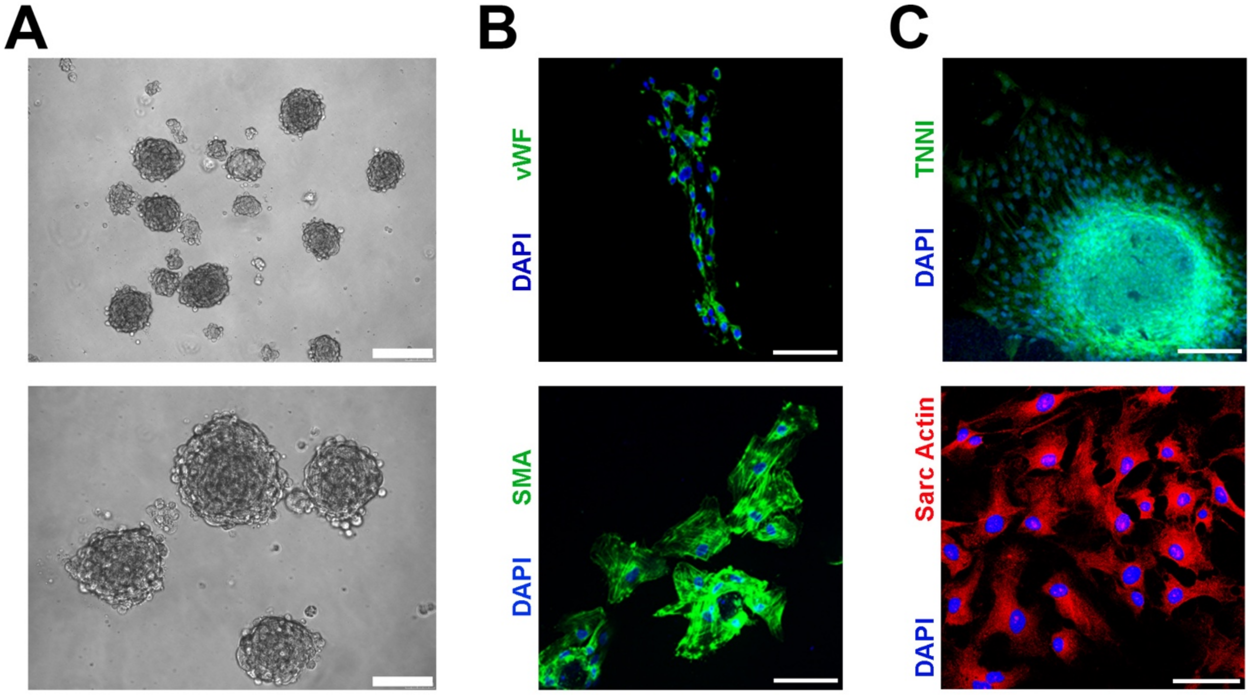

Fig. S3. Multilineage Differentiation Potential of human c-kit^{pos}/CD45^{neg}/CD31^{neg} CSCs. (A) Representative light microscopy image at two different magnifications of cardiospheres derived from c-kit^{pos}/CD45^{neg}/CD31^{neg} hCSCs. (Representative of n=6 biological replicates). Scale bar=200µm and 100 µm. **(B,C)** Representative confocal microscopy images of multipotent c-kit^{pos}/CD45^{neg}/CD31^{neg} hCSCs. These population of cells were able to efficiently differentiate into endothelial cells (vWF, green) and smooth muscle (SMA, green) cell lineages. Cardiospheres generated from clonal c-kit^{pos}/CD45^{neg}/CD31^{neg} hCSCs plated in cardiac differentiation media efficiently commit to cardiomyogenic cell lineages (TNNI, green; α-SA, red). Nuclei are stained in blue (DAPI). (Representative of n=6 biological replicates). Scale bar=100µm and 50µm.

Supplementary Figure 4

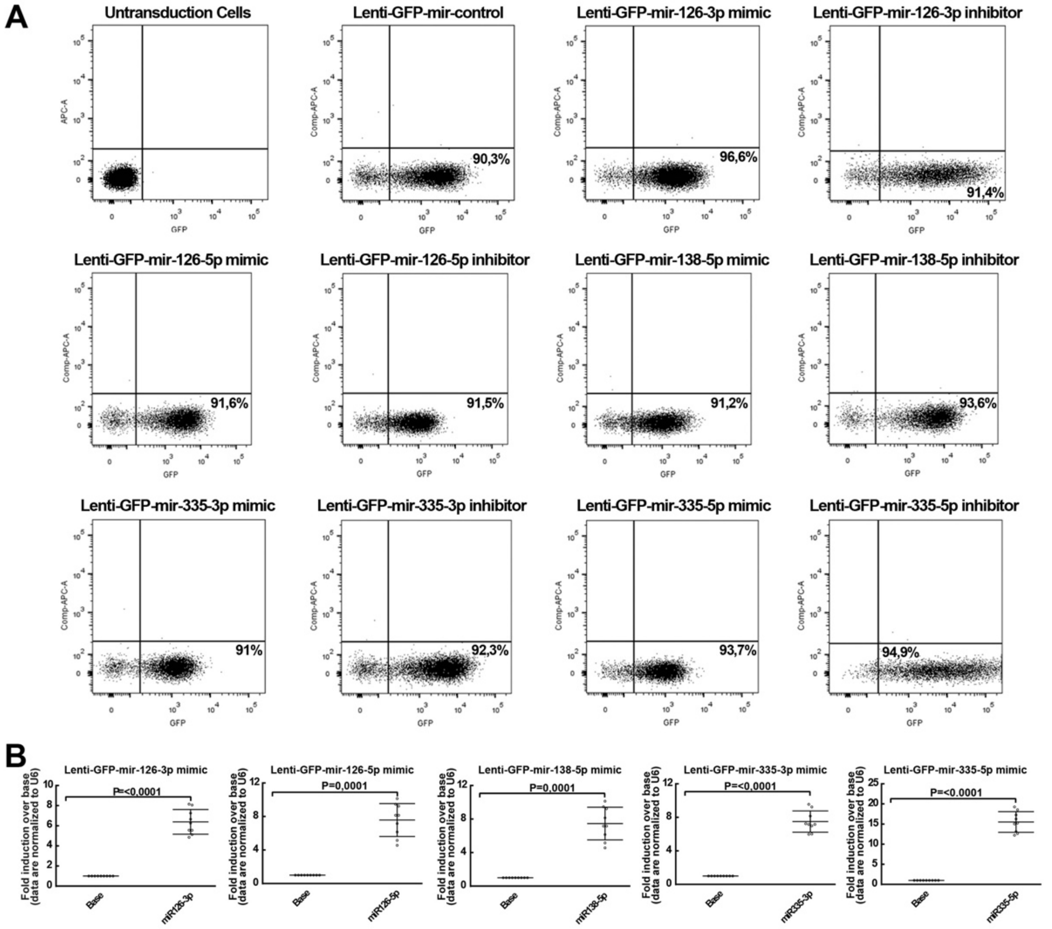

C

Gene	RC_norm_hCSCs	RC_norm_Myx-CSCs	Fold-Change	pval	padj
ANGPT1	412.71	54.53	-7.568	0.000000985	0.0000487
PLXNB2	1614.68	502.67	-3.212	0.0000138	0.0004102
S1PR3	240.21	117.24	-2.049	0.000662542	0.008286377
FBXL2	158.46	83.10	-1.907	0.004732524	0.034131361
BDNF	773.31	466.90	-1.656	0.006191666	0.041120693

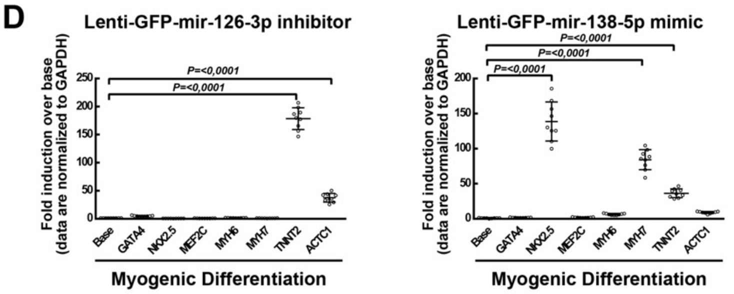

Fig. S4. microRNA modulation of the expansion/clonogenic deficit and abortive myogenic differentiation potential of c-kit^{pos}/CD45^{neg} /CD31^{neg} Myxoma-derived Cardiac Stem Cells *in vitro.* **(A)** Representative FACS dot plots showing the high efficiency of infection of the different Lentiviral vectors transducing GFP alone (control) or GFP together with the indicated specific miR-mimic or -inhibitor. **(B)** Graph showing RT-PCR data of specific microRNA upregulation by the respective Lentivirus miR-mimic-GFP infection*. **(C) Snapshot table** of regulated miR-126-3p mRNA targets in c-kit^{pos}/CD45^{neg} /CD31^{neg} Myxoma-derived Cardiac Stem Cells (MyxCSCs) vs. normal hCSCs. **(D)** Graphs showing qRT-PCR data of the expression levels of the indicated cardiac

genes in c-kit^{pos}/CD45^{neg} /CD31^{neg} myxoma-derived Cardiac Stem Cells infected with Lenti-miR-128-3p-Inhibitor and Lenti-miR-138-5p-Mimic after 14 days in cardiomyogenic media *in vitro*. Data are presented as fold induction over respective baseline values (Base).
