# Supplementary material for: Atrial myxomas arise from multipotent cardiac stem cells
Source: Eur Heart J. 2020 Apr 24;41(45):4332–45. doi: 10.1093/eurheartj/ehaa156 (PMC7735815; doi:10.1093/eurheartj/ehaa156)
Supplement: ehaa156_Supplementary_Data [file ehaa156_supplementary_data.zip › ehaa156_Suppl_data/Online Table 1.pdf]

Table 1A. Clinical data and pathological findings in 23 cases of cardiac myxoma

PATIENT No.	SEX	AGE	PRIMARY SITE	MACROSCOPIC APPEARANCE
1	FEMALE	60	Left atrial myxoma	Polypoid, irregular surface
2	FEMALE	58	Left atrial myxoma	Polypoid, irregular surface
3	FEMALE	52	Left atrial myxoma	Polypoid, irregular surface
4	MALE	63	Left atrial myxoma	Polypoid, irregular surface
5	MALE	64	Left atrial myxoma	Polypoid, smooth
6	FEMALE	51	Right atrial myxoma	Polypoid, irregular surface
7	MALE	82	Left atrial myxoma	Polypoid, irregular surface
8	FEMALE	56	Left atrial myxoma	Polypoid, smooth
9	MALE	67	Left atrial myxoma	Polypoid, irregular surface
10	MALE	71	Left atrial myxoma	Polypoid, irregular surface
11	FEMALE	37	Left atrial myxoma	Polypoid, smooth
12	MALE	66	Left atrial myxoma	Polypoid, smooth
13	MALE	80	Right atrial myxoma	Polypoid, irregular surface
14	FEMALE	60	Left atrial myxoma	Polypoid, irregular surface
15	MALE	53	Left atrial myxoma	Polypoid, irregular surface
16	FEMALE	60	Left atrial myxoma	Polypoid, irregular surface
17	FEMALE	62	Right atrial myxoma	Polypoid, irregular surface
18	FEMALE	53	Left atrial myxoma	Polypoid, irregular surface
19	FEMALE	47	Left atrial myxoma	Polypoid, irregular surface
20	FEMALE	41	Left atrial myxoma	Polypoid, irregular surface
21	FEMALE	59	Left atrial myxoma	Polypoid, smooth
22	FEMALE	71	Right atrial myxoma	Polypoid, irregular surface
23	FEMALE	61	Left atrial myxoma	Polypoid, irregular surface

Table 1B. Clinical and Surgical data of 10 control human samples.

PATIENT No.	SEX	AGE	PRIMARY SITE	SURGICAL INTERVENTION
1	MALE	67	Right Atrium	Mitral valve replacement
2	MALE	66	Right Atrium	CABG
3	MALE	58	Right Atrium	CABG
4	MALE	67	Right Atrium	CABG
5	MALE	57	Right Atrium	Aortic valve replacement
6	MALE	69	Right Atrium	Aortic valve replacement
7	FEMALE	61	Right Atrium	Aortic and mitral valve replacement
8	MALE	57	Right Atrium	CABG
9	FEMALE	68	Left Atrium	Aortic valve replacement
10	MALE	64	Right Atrium	Aortic valve replacement
