# Supplementary material for: Atrial myxomas arise from multipotent cardiac stem cells
Source: Eur Heart J. 2020 Apr 24;41(45):4332–45. doi: 10.1093/eurheartj/ehaa156 (PMC7735815; doi:10.1093/eurheartj/ehaa156)
Supplement: ehaa156_Supplementary_Data [file ehaa156_supplementary_data.zip › ehaa156_Suppl_data/Online Table 4.pdf]

Table 4. Antibodies' List

**Antigen**	**Antibody ID**	**Company**	**Application**
c-kit	A3C6E2	Miltenyi Biotec	FC
CD184 (CXCR4)	12G5	Miltenyi Biotec	FC
CD9	SN4 C3-3A2	Miltenyi Biotec	FC
CD44	DB105	Miltenyi Biotec	FC
CD63	H5C6	Miltenyi Biotec	FC
CD81	5A6	BioLegend	FC
CD90	DG3	Miltenyi Biotec	FC
CD166	3A6	BioLegend	FC
CD105	43A4E1	Miltenyi Biotec	FC
MDR-1	UIC2	BioLegend	FC
CD324 (E-CADHERIN)	67A4	Miltenyi Biotec	FC
PDGFR-α	16A1	BioLegend	FC
CD150	REA151	Miltenyi Biotec	FC
CD31	AC128	Miltenyi Biotec	FC
CD45	REA747	Miltenyi Biotec	FC
CD34	AC136	Miltenyi Biotec	FC
Mouse IgG1 - Isotype control antibody		Miltenyi Biotec	FC
Mouse IgG2a - Isotype control antibody		Miltenyi Biotec	FC
Mouse IgG2b - Isotype control antibody		Miltenyi Biotec	FC
Rat IgG1- Isotype control antibody		Miltenyi Biotec	FC
c-kit	H-300	Santa Cruz Biotech	IF
Oct-4	C-10	Santa Cruz Biotech	IF
Oct-4	AF1759	R&D system	IF
Nanog	PA1-097	PIERCE, Invitrogen	IF
Bmi-1	H99	Santa Cruz Biotech	IF
Nkx2.5	A-3	Santa Cruz Biotech	IF
Nkx2.5	AF2444	R&D system	IF
Islet-1	AF1837	R&D system	IF
vWF	F3520	Sigma-Aldrich	IF
SMA	1A4	Sigma-Aldrich	IF
CD31	WM-59	Sigma-Aldrich	IF
CD45	OX30	Santa Cruz Biotech	IF
Calretinin	Ab92341	Abcam	IF
Sarcomeric Actin	5C5	Sigma	IF
TNNI	Ab47003	Abcam	IF
Tryptase	Ab2378	Abcam	IF
GFP	600-101-215	Rockland	IF
Chondroitin Sulfate	CS-56	Sigma-Aldrich	IF

FC denotes Flow Cytometry; IF denotes cell immunofluorescence
