# Supplementary material for: Atrial myxomas arise from multipotent cardiac stem cells
Source: Eur Heart J. 2020 Apr 24;41(45):4332–45. doi: 10.1093/eurheartj/ehaa156 (PMC7735815; doi:10.1093/eurheartj/ehaa156)
Supplement: ehaa156_Supplementary_Data [file ehaa156_supplementary_data.zip › ehaa156_Suppl_data/Online Table 5 NEW.pdf]

Atrial Myxomas Arise From Multipotent Cardiac Stem Cells

Scalise M, Torella M, et al.

Table 5. RT-PCR Taqman Primers List

GAPDH	Hs02758991_g1
Oct-4	Hs04260367_gH
Nanog	Hs04260366_g1
Tert	Hs00972656_m1
Nkx2.5	Hs00231763_m1
Gata4	Hs00171403_m1
Bmi1	Hs00995519_g1
CD133	Hs01009259_m1
c-kit	Hs00174029_m1
CD45	Hs04189704_m1
CD34	Hs00990732_m1
CD31	Hs00169777_m1
vWF	Hs01109446_m1
Mdr-1	Hs00184500_m1
Acta2	Hs01009259_m1
Myh11	Hs00975796_m1
Tnnt2	Hs00165960_m1
Actc1	Hs01109515_m1
hsa-miR-335	000546
hsa-miR-335-3p	002185
hsa-miR-126-3p	002228
hsa-miR-126-5p	000451
hsa-miR-138-5p	002284
