# Supplementary material for: Atrial myxomas arise from multipotent cardiac stem cells
Source: Eur Heart J. 2020 Apr 24;41(45):4332–45. doi: 10.1093/eurheartj/ehaa156 (PMC7735815; doi:10.1093/eurheartj/ehaa156)
Supplement: ehaa156_Supplementary_Data [file ehaa156_supplementary_data.zip › ehaa156_Suppl_data/Online Table 6 NEW.pdf]

Atrial Myxomas Arise From Multipotent Cardiac Stem Cells

Scalise M, Torella M, et al

Table 6. miRNA Lentivirus list

Cat. No.	Name
m002	pLenti-III-mir-GFP-Blank
mh16573	LentimiRa-GFP-hsa-miR-126-5p Virus
mh15089	LentimiRa-GFP-hsa-miR-126-3p Virus
mh15177	LentimiRa-GFP-hsa-miR-138-5p Virus
mh15471	LentimiRa-GFP-hsa-miR-335-5p Virus
mh16766	LentimiRa-GFP-hsa-miR-335-3p Virus
mh35100	LentimiRa-Off-hsa-miR-126-5p Virus
mh35099	LentimiRa-Off-hsa-miR-126-3p
mh35179	LentimiRa-Off-hsa-miR-138-5p
mh35549	LentimiRa-Off-hsa-miR-335-5p
mh35550	LentimiRa-Off-hsa-miR-335-3p
